# Mesenchymal stem cells: a novel therapeutic approach for feline inflammatory bowel disease

**DOI:** 10.1186/s13287-024-04038-y

**Published:** 2024-11-09

**Authors:** Qiyun Xie, Saisai Gong, Jintao Cao, Aoyun Li, Md. F. Kulyar, Bingyun Wang, Jiakui Li

**Affiliations:** 1 College of Veterinary Medicine, Huazhong Agricultural University, Wuhan, 430070 P.R. China; 2 School of Life Science and Engineering, Foshan University, Foshan, P.R. China; 3 College of Veterinary Medicine, Henan Agricultural University, Zhengzhou, P.R. China

**Keywords:** Inflammatory bowel disease, Cat model, Mesenchymal stem cells (MSCs), Intestinal microbiota

## Abstract

**Background:**

Inflammatory bowel disease (IBD) poses a significant and growing global health challenge, affecting both humans and domestic cats. Research on feline IBD has not kept pace with its widespread prevalence in human populations. This study aimed to develop a model of feline IBD by incorporating dextran sulfate sodium (DSS) to evaluate the therapeutic potential of MSCs and to elucidate the mechanisms that enhance their action.

**Methods:**

We conducted a comprehensive clinical assessment, including magnetic resonance imaging (MRI), endoscopy, and histopathological examination. Additionally, alterations in intestinal microbiota were characterized by 16 S rDNA sequencing, and the influence of MSCs on IBD-related gene expression was investigated through transcriptome analysis.

**Results:**

According to our findings, MSC treatment significantly mitigated DSS-induced clinical manifestations, reduced inflammatory cell infiltration, decreased the production of inflammatory mediators, and promoted mucosal repair. Regarding the intestinal microbiota, MSC intervention effectively corrected the DSS-induced dysbiosis, increasing the presence of beneficial bacteria and suppressing the proliferation of harmful bacteria. Transcriptome analysis revealed the ability of MSCs to modulate various inflammatory and immune-related signaling pathways, including cytokine-cytokine receptor interactions, TLR signaling pathways, and NF-κB pathways.

**Conclusion:**

The collective findings indicate that MSCs exert multifaceted therapeutic effects on IBD, including the regulation of intestinal microbiota balance, suppression of inflammatory responses, enhancement of intestinal barrier repair, and modulation of immune responses. These insights provide a solid scientific foundation for employing MSCs as an innovative therapeutic strategy for IBD and pave the way for future clinical explorations.

**Supplementary Information:**

The online version contains supplementary material available at 10.1186/s13287-024-04038-y.

## Background

Inflammatory bowel disease (IBD) is a common chronic gastrointestinal disorder that includes ulcerative colitis (UC) and Crohn’s disease (CD). Its etiology and exact pathogenesis remain unknown [1]. Chronic autoimmune disease (IBD) is primarily characterized by intestinal inflammation, and the root cause is typically thought to be an aberrant immune response of genetically predisposed hosts to gut microbiota influenced by genetic abnormalities and external triggers [2]. At the site of inflammation, intestinal myofibroblasts are activated and upregulate a range of cytokines, chemokines, growth factors, and adhesion molecules, increasing the secretion of soluble mediators and extracellular matrix factors that promote inflammation [3]. This response is markedly different from that of a healthy gut, where intestinal myofibroblasts remain static and inactive. The pathogenesis of IBD may be related to dysfunction in gut microbiota and the intestinal mucosal immune system; abnormal T cell function or cytokine imbalances may disrupt the intestinal mucosal immune system [4]. In normal intestines, effector and regulatory immune cells mainly stability by regulating one another through a complex cytokine network [2]. In the inflamed gut, the lack of a regulatory immune response leads to inflammation of the intestinal mucosa and disrupts the homeostasis of the intestinal mucosal immune system [4]. The exact etiology of feline IBD is not fully understood, but it is believed to involve a complex interplay of genetic predisposition, environmental factors, and dysregulation of the immune system [5]. Current treatment options for feline IBD primarily focus on managing and reducing symptoms through dietary modifications, antibiotics, corticosteroids, and immunosuppressive agents [6]. However, these treatments are often associated with limited efficacy, side effects, and potential long-term complications. Currently, the international diagnosis of cat inflammatory bowel disease is limited. It can only be suggested but not confirmed based on medical history, clinical symptoms, hematology, and imaging. Colonoscopy biopsies may be missed or misdiagnosed, and the final diagnosis requires a full-thickness colon biopsy, which is difficult to obtain clinically [7].

The balance of the gut microbiota is closely related to the etiology of IBD. Empirical evidence has underscored the pivotal role of the gut microbiota’s composition and functionality in the pathogenesis and progression of IBD [8]. The gut microbiota modulates the integrity of the intestinal barrier and the activity of immune cells by interacting with the host’s immune system. In the context of IBD, reduced diversity of the gut microbiota is characterized by an increase in potentially pathogenic bacteria and an overall reduction in beneficial bacteria. This results in a weakened intestinal barrier and an intensified inflammatory response [9].

Furthermore, the gut microbiota is a source of essential metabolites, such as short-chain fatty acids, crucial in regulating the host’s immune response and maintaining gut health [10]. Consequently, interventions aimed at restoring the balance of the gut microbiota have emerged as a novel therapeutic strategy for IBD. Approaches such as probiotics, prebiotics, and fecal microbiota transplantation (FMT) have demonstrated efficacy in enhancing the diversity and functionality of the gut microbiota, thereby improving the clinical manifestations of IBD [11].

Due to their distinctive biological attributes, mesenchymal stem cells (MSCs) have garnered considerable attention in regenerative medicine as a therapeutic agent for IBD. These cells can mitigate intestinal inflammation by secreting a range of anti-inflammatory cytokines and growth factors, including IL-10 and TGF-β [12]. Furthermore, MSCs have been shown to promote the regeneration and repair of intestinal epithelial cells, thereby enhancing the intestinal barrier function [13]. A significant aspect of MSCs’ influence lies in their regulatory impact on the intestinal microbiota. Recent research has indicated that MSCs can modulate the composition of the intestinal microbiota, promoting the proliferation of beneficial bacteria and suppressing the growth of pathogenic species, which helps to re-establish the equilibrium of the microbiota [14]. These insights have illuminated new avenues for the therapeutic application of MSCs in IBD. As we progress, upcoming research efforts must explore the complex mechanisms of the interplay between MSCs and the intestinal microbiota. A comprehensive understanding of this interaction is essential for developing effective treatment strategies for IBD. Clarifying these mechanisms could pave the way for innovative therapeutic approaches that leverage the synergistic effects of MSCs and the modulation of the intestinal microbiota. The present study utilized a DSS-induced model to replicate feline IBD-like intestinal injury and dysbiosis. The objective was to investigate the therapeutic efficacy of MSC treatment on feline IBD and to clarify its underlying mechanisms of action. Moreover, we aimed to assess the potential therapeutic benefits of this novel approach in managing IBD.

## Materials and methods

### Materials

Dextran sulfate sodium salt (DSS, M.W 40000) was purchased from Shanghai Macklin Reagent Biochemical Technology Co., Ltd. Cat-derived mesenchymal stem cells (MSCs) were provided by Vet Cell Biotechnology Co., Ltd. Antibodies were supplied by Powerful Biology (Wuhan) Co., Ltd. and ELISA kits were purchased from ABclonal Technology (Wuhan) Co., Ltd. and Primer design and synthesis were performed by Sangon Biotech (Shanghai) Co.

### Animal experiment design

Ten adult short-haired cats with similar body weights were obtained from the Huazhong University Laboratory Animal Center and divided into the control group (C) and the DSS drinking water group (D). Following a week of acclimatization, cats in group D were given 3% DSS drinking water for one week, followed by a week of regular drinking water, repeating this cycle for four weeks. Cats in group C received normal drinking water throughout the experiment. The weights and fecal scores of the cats were recorded regularly. After four weeks, magnetic resonance imaging and endoscopy of the intestines were conducted. Colon tissue samples were surgically collected for histopathologic examination, with two group D samples randomly selected for evaluation at the Shouqiu Reference Laboratory (SQ Lab). Fifteen adult short-haired cats were equally divided into the control group (C), DSS drinking water group (D), and mesenchymal stem cell group (M). Cats in group M received weekly intravenous injections of MSCs (10^^6^ cells/mL/kg) from week 5 to week 8, while groups C and D were given normal saline as a negative control. Throughout the study period, the weight and fecal scores of the cats were documented.

At the end of the eighth week, serum samples were collected at -20 ℃ for cytokine detection and evaluation of intestinal injury using magnetic resonance imaging and endoscopy. Colon tissue and fecal samples were surgically obtained, with some fixed in 4% paraformaldehyde and others stored at -80 ℃ after rapid cooling with liquid nitrogen. All cats in the experiment were provided with a comfortable environment, along with ample food and water. All samples were collected in the sterile operating room of the Animal Hospital affiliated with Huazhong Agricultural University, with collaboration from professional surgeons and anesthetists. The participating cats underwent partial colectomy under conditions that did not compromise their subsequent quality of life, and no fatalities occurred during the procedure. Postoperative care for all cats was managed by the nursing department. All animal experiments were conducted in accordance with the Guidelines for the Care and Use of Laboratory Animals and approved by the Ethics Committee of the Laboratory Animal Center of Huazhong Agricultural University (HZAUCA-2024-0026) and the ARRIVE (Animal Research: Reporting of In Vivo Experiments) 2.0 guidelines.

### Magnetic resonance image analysis

Before the experiment, the cats underwent a 12-hour fast to empty the colon and reduce peristalsis. Anesthesia was induced using a combination of 4 mg/kg of telazol (tiletamine/zolazepam (Virbac S.A., France) and 0.01 mg/kg of dexmedetomidine (Orion Pharma, Finland) and maintained with 0.5-2% isoflurane (Shenzhen Rayward Life Technology Co., Ltd). Vital signs, including heart rate, respiratory rate, and oxygenation, were monitored throughout the procedure. The cat was positioned on the magnetic resonance imaging bed, with a dedicated receiver-only coil placed above the abdomen to optimize image quality. Axial T2-weighted imaging (T2WI) sequences were performed using a 1.5-tesla magnetic resonance imaging scanner. The image acquisition parameters included a TR of 5100 ms, TE of 100 ms, slice thickness of 3 mm, matrix size of 220 × 220, and a field of view (FOV) of 20 cm x 20 cm. The acquired T2WI images were then transferred to a specialized post-processing workstation for analysis. The thickness of the colon wall was evaluated and measured by three veterinary radiologists who maintained the clinical information.

### Endoscopic observation

Before the procedure, the cat underwent a 12-hour fast to empty the colon and prepare for an endoscopy. Anesthesia was administered using a combination of 4 mg/kg of tiletamine/zolazepam (Virbac S.A., France) and 0.01 mg/kg of dexmedetomidine (Orion Pharma site, Finland), with maintenance on 0.5-2% isoflurane (Shenzhen Rayward Life Technology Co., Ltd). Vital signs, including heart rate, respiratory rate, and oxygenation, were monitored throughout the process. A colonoscopy was performed using a flexible video endoscope equipped with a light source and a high-resolution camera. The endoscope was carefully inserted through the anus into the colon under direct visualization. The camera and light direction were adjusted to observe the colonic mucosa in real-time and capture images. In cases where a significant amount of feces remained in the intestinal cavity, a warm, normal saline enema was necessary.

### Histopathological analysis

The intestinal samples were fixed in a 4% paraformaldehyde solution for 24 h. Subsequently, the samples were embedded in paraffin and sectioned. These sections were then stained with hematoxylin and eosin to enable observation and examination of tissue morphology.

### ELISA

Levels of IL-1β, TNF-α, and IL-6 in serum samples were measured using a commercial ELISA kit obtained from ABCLONAL Technology Co., Ltd. Briefly, diluted serum, and standard samples were incubated in coated wells, followed by the addition of specific HRP markers to detect antibodies. After washing, a TMB substrate was added, resulting in a blue color that turned yellow upon adding an acid terminator. The color intensity correlated with the amount of the target antibody in the sample. The optical density (OD) was measured using a microplate reader at 450 nm, and the concentration of the target antibody in the sample was determined by comparing the OD value to the established standard curve.

### Immunohistochemistry

Colon tissue samples were fixed in a 4% paraformaldehyde solution for 24 h and embedded in paraffin. Serial sections of 4 μm thickness were cut from each tissue block using a microtome. These sections underwent dewaxing in xylene and rehydration through an ethanol series. The slices were placed in a citric acid buffer (pH 6.0) and subjected to a 15-minute heat treatment in a pressure cooker to aid antigen recovery. Next, the sections were exposed to 3% hydrogen peroxide for 10 min to block endogenous peroxidase activity before being incubated with primary antibodies overnight at 4 °C. The optimal dilution of primary antibodies was determined according to the manufacturer’s instructions. After the sections were washed with phosphate-buffered saline (PBS), they were incubated with horseradish peroxidase secondary antibody for 1 h at room temperature. Sections were developed using 3,3’-diaminobenzidine (DAB) as a developer and then counterstained with hematoxylin. Overall, this immunohistochemical analysis enabled reliable detection and quantification of the target protein in the tissue sections, providing crucial information for further research and potential clinical applications.

### Western blot

Following the immediate retrieval of colon tissue samples from − 80 °C, protein lysis treatment was conducted in RIPA buffer containing protease and phosphatase inhibitors using a tissue grinder. The protein concentration in the supernatant was determined through enhanced BCA protein analysis. Subsequently, equal quantities of protein samples were separated via SDS-PAGE gel electrophoresis. The isolated proteins were transferred onto PVDF membranes using a Bio-Rad transfer apparatus. These membranes were blocked with TBST buffer containing 5% skim milk powder, followed by an overnight incubation at 4 °C with primary antibodies targeting the protein of interest and β-actin (a loading control). After washing the membranes with TBST buffer, HRP-bound secondary antibodies were incubated for 1 h at room temperature. Protein bands were visualized using a chemiluminescent (ECL) substrate and captured with the Bio-Rad ChemiDoc XRS + system. Band intensities were quantified using ImageJ software, and the protein expression levels were normalized against β-actin levels.

### Microflora analysis of colon contents

Bacterial DNA was extracted from fecal particles using the DNeasy PowerSoil kit (Qiagen, Hilden, Germany). Primers designed according to the V3-V4 conserved region of the bacterial 16 S rRNA gene (343 F: 5’-TACGGRAGGCAGCAG-3’; 798R: 5’-AGGTATCTAATCCT-3’) were amplified by PCR and the products were purified, quantified and homogenized to form sequencing libraries, which were then sequenced using the Illumina NovaSeq 6000. Quality control of the original sequencing data, including low-quality and length filtering, was performed to obtain high-quality sequences. The high-quality sequences were clustered and de-noised; operational taxonomic units (OTUs) were delineated, and a Venn diagram was used to show the distribution of feature numbers among the groups. With SILVA as the reference database, the naive Bayes classifier was used to make taxonomic annotations on the feature sequences, from which species classification information corresponding to each feature could be obtained, and the sample community composition was analyzed. QIIME software was used to generate species abundance at different classification levels and to create a column chart of species distribution between groups. The species diversity within a single sample was assessed using alpha diversity analysis, with the Ace, Chao1, Shannon, and Simpson indices calculated for each sample, along with the generation of sample dilution curves and grade abundance curves. Beta diversity analysis was conducted to compare species diversity among different samples, represented by a sample level clustering (UPGMA) tree and a PCoA score map. To evaluate the differences in microbial community abundance between study groups, a T-test was performed on species abundance data using Metastats software. P-values were obtained, allowing for the identification of species with significant differences in sample composition between the groups; a histogram was generated using GraphPad Prism version 7.0c (GraphPad Software, Inc., California, USA). Based on abundance and variation of each species in each sample, Spearman rank correlation analysis was conducted, and data with a correlation greater than 0.1 and a p-value less than 0.05 were selected to construct a correlation network to further elucidate the mechanisms underlying phenotypic differences between samples.

### Transcriptome analysis of colon tissue

Total RNA from the colon was isolated using Trizol reagents (Invitrogen Life Technologies), followed by concentration, quality, and integrity measurements using a NanoDrop spectrophotometer (Thermo Scientific). In the Illumina Hi-Seq sequencing platform (cBot, Illumina), quality-controlled RNA samples were sequenced on the Hi Seq X ten system. Image recognition, decontamination, and decoupling were performed on the sequencing results, and the number of sequencing reads, data yield, sequencing error rate, Q20 content, Q30 content, and GC content were quantified. The distribution of sequencing bases A, T, C, G, and reads along the 5 ' to 3’ direction as analyzed to evaluate sequencing accuracy. Meanwhile, the difference between the actual and theoretical distributions of GC content was compared. PCA score maps and inter-sample correlation heat maps were used to analyze the similarity of expression patterns between samples and groups. The R language package DESeq2 was employed for differential analysis, and differentially expressed genes with a threshold of false discovery rate (FDR) < 0.05 were identified. The inter-group volcano plot of differential gene expression was generated based on the distribution of FDR and fold change (FC) values among samples. The KEGG database was utilized to identify the pathways in which differentially expressed genes were significantly enriched relative to all annotated genes through pathway significance enrichment, and bubble maps for KEGG enrichment analysis of differentially expressed genes were created. The differentially expressed genes involved in critical biochemical and metabolic pathways as well as signal transduction pathways, were statistically analyzed, and the relationship between pathways and gene networks was mapped.

### Correlation analysis

OriginPro 2021 software was used to analyze the relationship between gut microbiota abundance and the gene expression of inflammatory markers by calculating the Pearson correlation coefficient and generating a correlation heat map.

### Statistical analysis

Values are expressed as mean ± standard deviation. Univariate analysis of variance and Tukey’s multiple range test were used to compare groups. Significance was defined as *p* < 0.05. Statistical analysis was conducted using SPSS (v21.0) software, and charts were created with GraphPad Prism (v7.0) software.

## Results

### Evaluation of experimental cat IBD model

**Fig. 1:**
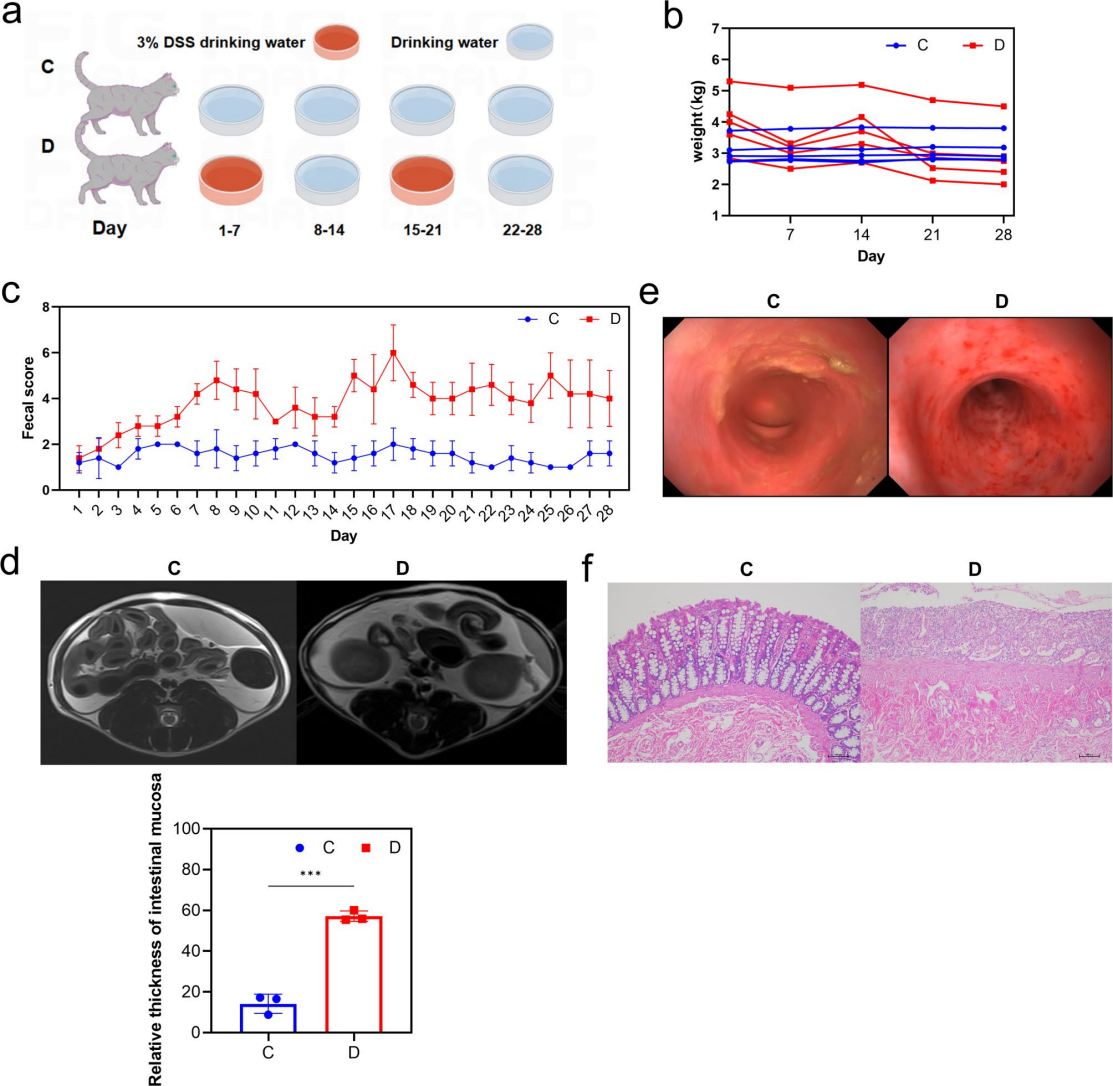
Construction and evaluation of a feline IBD Model. (**a**) Procedure for the induction of feline IBD-like injury; (**b**) Body weight monitoring over the experimental period; (**c**) Fecal scoring system to assess intestinal inflammation; (**d**) Endoscopic images of the colon showcasing mucosal changes; (**e**) Magnetic resonance imaging scans of the colon with measurements of mucosal thickness; (**f**) Histopathological images of colonic tissue illustrating the extent of injury and inflammation. Significant variations had been denoted with * (*p* < 0.05), ** (*p* < 0.01), or *** (*p* < 0.001)

A feline IBD model was developed by incorporating 3% dextran sulfate sodium (DSS) into the drinking water of the cats (Fig. 1a). Over four weeks, the IBD induction regimen was implemented during the first and third weeks. Our findings revealed a consistent decline in body weight, indicating an adverse response to DSS (Fig. 1b). Throughout the observational period, fecal scores for the DSS-exposed group were significantly elevated compared to the control group (Fig. 1c). Using magnetic resonance imaging on day 28, we observed a pronounced thickening of the colonic wall in cats subjected to DSS treatment (Fig. 1d). Endoscopic examination further revealed significant hemorrhaging and ulcerations within the colonic mucosa of the DSS-treated cats (Fig. 1e). Additionally, histological examination (HE staining) indicated a substantial loss of the typical mucosal architecture in the DSS group, characterized by extensive necrosis, ulceration, and pronounced inflammatory cell infiltration (Fig. 1f). These clinical manifestations and colonic pathologies resembled those observed in IBD. To substantiate these findings, anonymized samples were sent to a specialized pathology laboratory for an unbiased assessment, resulting in a diagnosis of lymphoplasmacytic colitis consistent with IBD features (refer to the Supporting Information for the complete pathology report). These observations confirm the successful establishment of the feline IBD model, which serves as a robust platform for subsequent investigative endeavors.

### Effect of MSC on clinical treatment of experimental cat IBD model

**Fig. 2:**
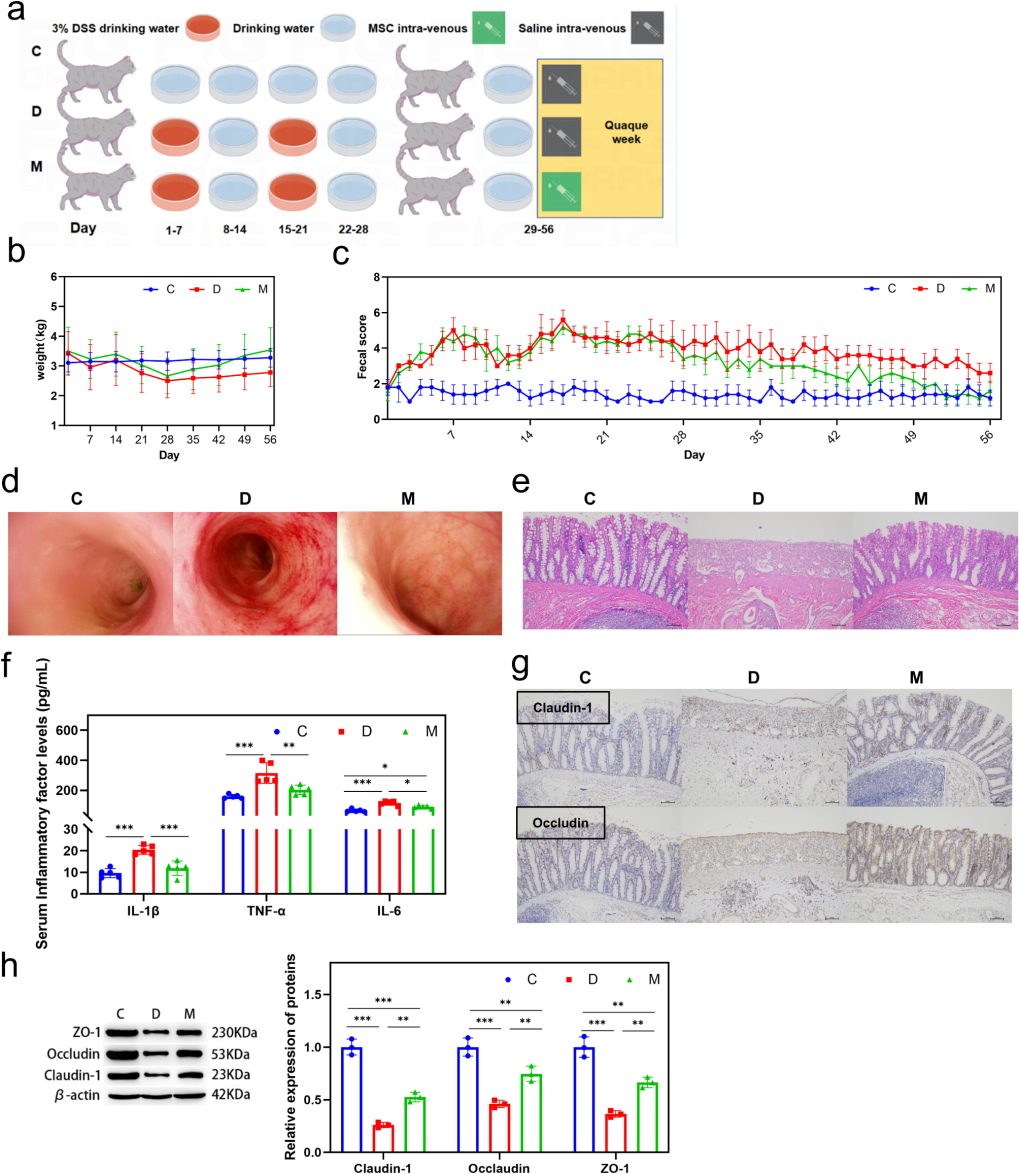
MSCs improve clinical signs of feline IBD. (**a**) Feline IBD-like injury treatment procedures; (**b**) Monitoring of body weight as an indicator of efficacy; (**c**) Assessment of fecal scores reflecting intestinal health; (**d**) Endoscopic observation of the colonic mucosa before and after MSC treatment; (**e**) Histopathological examination of colonic tissues after MSC intervention; (**f**) Quantitative analysis of serum levels of inflammatory cytokines; (**g**) Immunohistochemical staining of the colon showing MSCs’ protective effects of MSCs on the intestinal barrier; (**h**) Expression levels and relative quantification of colonic barrier proteins. Significant variations had been denoted with * (*p* < 0.05), ** (*p* < 0.01), or *** (*p* < 0.001)

A model was procured using the methodology mentioned above to elucidate the therapeutic efficacy of MSCs on feline IBD, which was subsequently employed for therapeutic evaluation. The treatment regimen commenced post-colonic injury, with MSCs administered weekly (Fig. 2a). After the intervention, MSCs effectively ameliorated weight loss and fecal irregularities attributed to colitis (Fig. 2b, c). After day 56, an endoscopic examination revealed that MSCs significantly aided in the repair of colonic abnormalities compared to the untreated group (Fig. 2d). Moreover, histological examination (HE staining) confirmed that MSCs restored the architectural integrity of the damaged colonic tissue (Fig. 2e). To explore the therapeutic implications of MSCs on feline colitis, the serum activity of inflammatory biomarkers and the structural status of the intestinal barrier were assessed. Enzyme-linked immunosorbent assay (ELISA) demonstrated that MSCs markedly attenuated the DSS-induced surge in pro-inflammatory cytokines IL-1β, TNF-α, and IL-6, thereby significantly mitigating the systemic inflammatory response (Fig. 2f). Immunohistochemical (IHC) and western blot analysis revealed that MSC treatment significantly enhanced the expression of Claudin-1 and Occludin, further validating the reparative effects of MSCs on the impaired intestinal barrier (Fig. 2g and h). In summary, the findings collectively demonstrate the pronounced therapeutic impact of MSCs on feline IBD.

### Reversal of DSS-induced gut microbiota dysbiosis by MSC and its potential role in the treatment of feline IBD

**Fig. 3:**
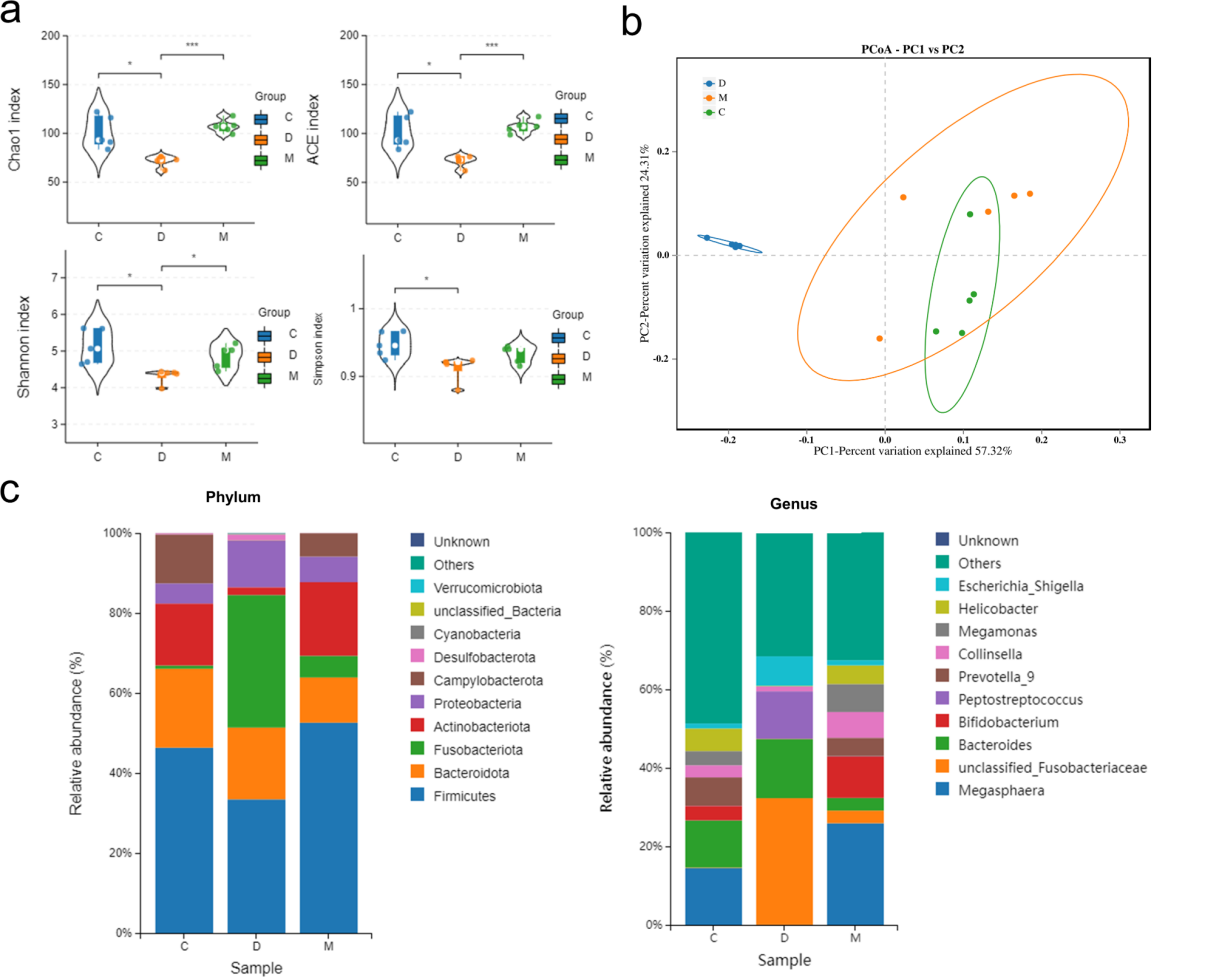
MSCs modulate gut microbiota composition changes induced by feline IBD-like Injury. (**a**) Alpha diversity indices of the gut microbiota; (**b**) Beta diversity indices illustrating variations among microbial communities; (**c**) Taxonomic composition at the phylum and genus levels within the bacterial communities

DSS-induced colonic injury is frequently associated with dysbiosis of gut microbiota. To elucidate the relationship between the therapeutic effects of MSCs on feline IBD and changes in gut microbiota, we sequenced the colonic content based on the conserved region of bacterial 16 S rDNA. To assess the overall microbial community richness and diversity, multiple α-diversity indices were measured. DSS treatment induced significant shifts in the feline colonic microbiota, evidenced by substantial declines in the Chao1, ACE, Shannon, and Simpson indices, indicating a reduction in microbial species and diversity. Notably, MSC treatment effectively reversed these effects, stabilizing the disturbed gut flora (Fig. 3a). Furthermore, β-diversity was calculated to evaluate differences among the gut microbial communities of the three groups. Principal Coordinate Analysis (PCoA) illustrated that MSC treatment mitigated the DSS-induced changes in colonic microbial composition (Fig. 3b). The use of a UPGMA phylogenetic tree to evaluate species composition similarity confirmed that cats treated with MSCs had colonic microbial profiles more similar to those of the control group (Fig. S2).

We evaluated the changes in the composition and relative abundance of dominant colonic bacteria following distinct treatment regimens (Fig. 3c). At the phylum level, there was a significant reduction in the proportion of Firmicutes (46.33%, 33.41%, and 52.58% in groups C, D, and M, respectively), Actinobacteriota (15.48%, 1.94%, and 18.42% in groups C, D, and M, respectively), and Campylobacterota (12.26%, 0.15%, and 5.85% in groups C, D, and M, respectively) post-DSS treatment. Furthermore, the abundance of Fusobacteriota, Proteobacteria, and Desulfobacterota was also significantly diminished. At the genus level, the DSS intervention resulted in a decrease in the prevalence of Megasphaera, Megamonas, Prevotella_9, Bifidobacterium, Collinsella, and Helicobacter, with respective abundances in groups C, D, and M. Conversely, the expression of Peptostreptococcus and Escherichia_Shigella was significantly upregulated in response to DSS treatment. MSCs induced a counteractive trend in the modulation of these gut microorganisms. The data indicate that DSS administration significantly perturbates the gut bacterial community’s diversity, composition, and abundance. Conversely, the intervention with mesenchymal stem cells (MSCs) appeared to facilitate recovery of the gut microbiota, guiding it towards a state of equilibrium similar to that observed before the DSS-induced disruption.

### Regulatory effects of MSC on the gut microbiota and their potential mechanisms in the treatment of IBD

**Fig. 4:**
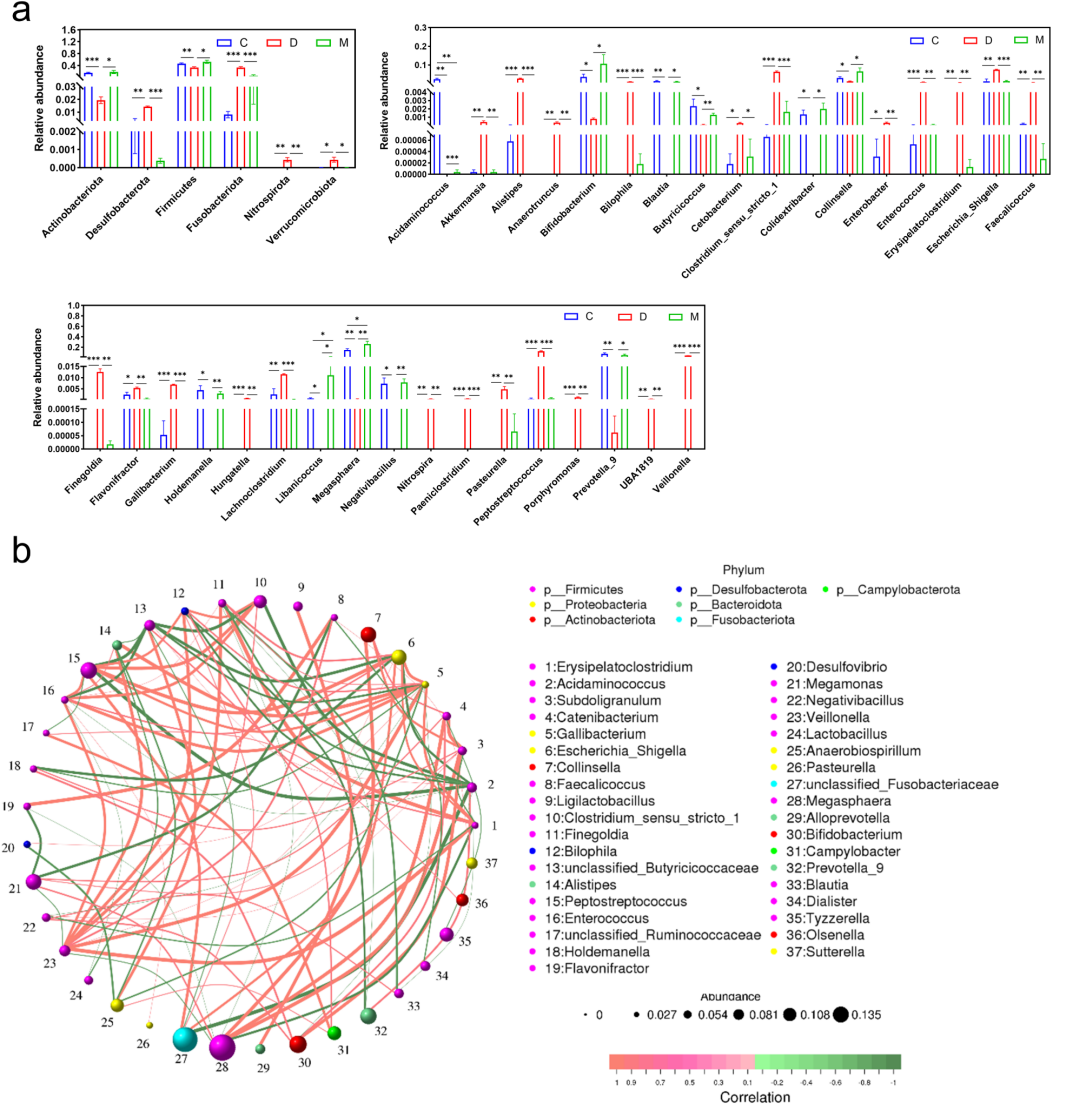
MSCs Modulate the Gut Microbiota to Ameliorate Feline IBD-like Injury. (**a**) Differential bacterial taxa at the phylum and genus levels; (**b**) Bacterial correlation network analysis. Significant variations had been denoted with * (*p* < 0.05), ** (*p* < 0.01), or *** (*p* < 0.001)

To elucidate the modulatory effects of MSCs on gut microorganisms, t-tests were conducted on species abundance data between treatments using Metastats software, thereby identifying marker species with potential biological significance (Fig. 4a). The analysis revealed that the phyla Actinobacteria and Firmicutes were significantly diminished following DSS intervention. At the same time, Desulfobacterota, Fusobacteriota, Nitrospirota, and Verrucomicrobiota were markedly enriched, with the latter being scarcely present in the healthy state. Notably, 34 bacterial genera exhibited significant changes in relative abundance post-DSS exposure, with 24 genera increasing and 10 decreasing. Strikingly, 11 genera were uniquely expressed under DSS conditions, including Anaerotruncus, Bilophila, and others, while six genera, such as Acidaminococcus and Blautia, were eliminated from the gut microbiota. These observations suggest that DSS induces intestinal damage and disrupts the gut microbiota. Intriguingly, MSC intervention substantially reversed the DSS-induced alterations in gut bacteria, implying a corrective capacity of MSCs on the disturbed gut microbiota. It is hypothesized that these bacteria may be pivotal in IBD development and crucial targets for MSC-mediated gut repair.

Spearman rank correlation networks were constructed to delineate species co-occurrence and interactions within the environmental samples to further investigate the role of gut microbial changes in MSC-mediated IBD treatment, revealing pattern information that is crucial for understanding phenotypic differences between samples (Fig. 4b). The results indicated that mutual regulation among bacteria was predominantly associated with Firmicutes, followed by Proteobacteria, which consistently accounted for over half of the intestinal microbial composition irrespective of treatments (Fig. 3c). This underscores their potential importance to the host. A significant finding was the negative correlation between bacteria enriched by DSS and those enriched by MSCs, along with a positive correlation with those inhibited by MSCs, suggesting a protective role of MSCs for bacteria suppressed by DSS and an inhibitory effect on those promoted by DSS. For instance, the inhibitory effect of DSS-enriched Gallibacterium on Bifidobacterium, Holdemanella, and Negativibacillus was mitigated by MSCs. Furthermore, MSCs significantly curbed the DSS-induced increase of Escherichia_Shigella and Peptostreptococcus, which were positively correlated with DSS-enriched bacteria. MSCs also reversed the DSS-induced decreases in Peptostreptococcus, Clostridium_sensu_stricto_1, and Escherichia_Shigella, and the elevation of Prevotella_9 resulting from the reduction of Acidaminococcus. The fact that MSCs promote Blautia, which should have declined along with Negativibacillus, provides further evidence of the complex interactions between intestinal microbes and regulatory networks involving MSC treatment.

### Transcriptome analysis reveals protective effects of MSC against IBD in cats

**Fig. 5:**
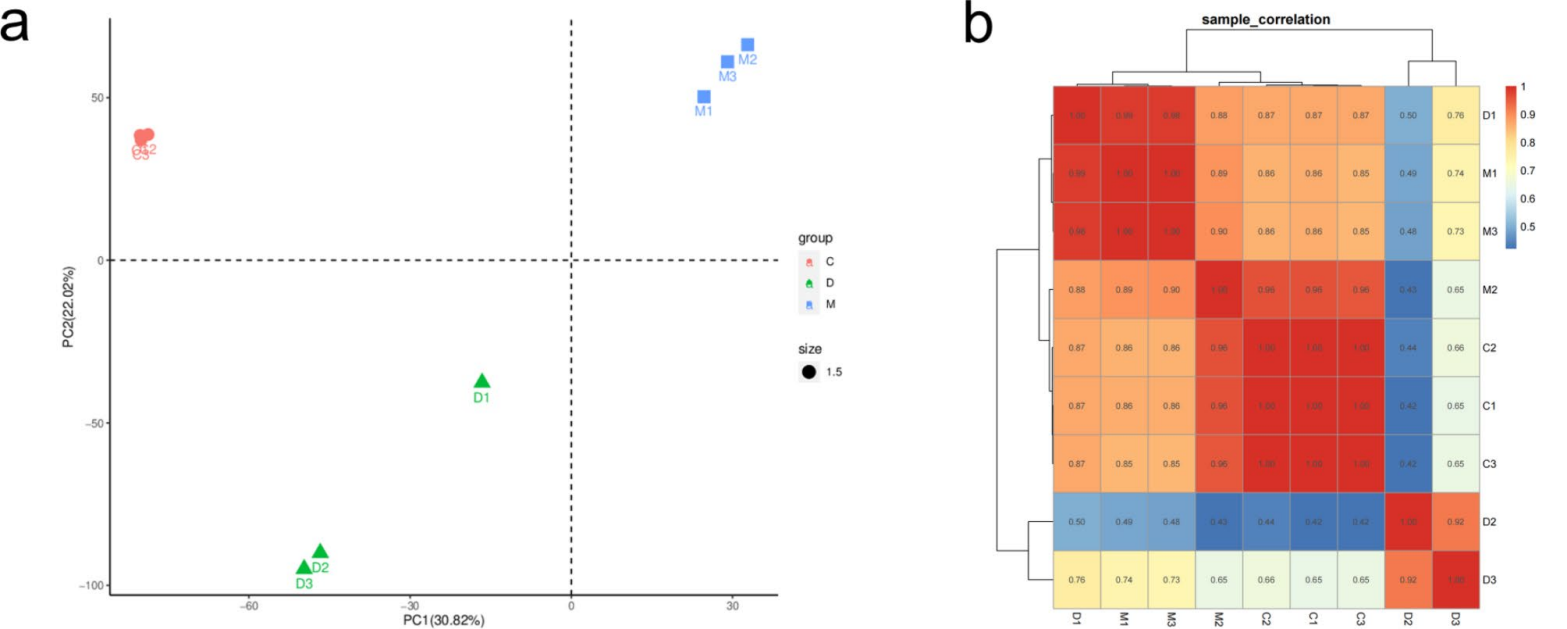
MSCs Ameliorate Intestinal Transcriptomic Alterations Induced by Feline IBD-like Injury. (**a**) PCA plot illustrating sample clustering; (**b**) Heatmap depicting sample correlation coefficients

A comprehensive transcriptome analysis of colonic tissues was conducted to further delineate the effects of MSCs in a feline model of experimental IBD. The raw read counts across the nine samples ranged from 36,507,238 to 59,482,248, as depicted in Figure S5a. All samples exhibited a uniform sequencing read length of 150 nucleotides, with Q20 ratios exceeding 90% and Q30 ratios ranging between 83% and 87%, averaging 85%. The GC content varied from 47 to 52% (Fig. S3a). An analysis of the base composition, sequencing quality, and GC content revealed a balanced distribution of A: T and C: G ratios, along with high sequencing quality and a GC content closely aligned with theoretical predictions (Fig. S3b-d). These metrics affirm the accuracy of the RNA-Seq data, rendering it suitable for subsequent analyses. Principal component analysis (PCA) distinguished distinct genetic profiles between DSS-challenged and MSC-treated colon tissues when compared to healthy controls (Fig. 5a). Correlation analyses between sample groups indicated varying degrees of similarity, with coefficients ranging from 0.42 to 0.99, underscoring the closer expression patterns between MSC-treated and control groups (Fig. 5b). This suggests that MSC treatment may mitigate the transcriptional perturbations induced by DSS.

### MSC influences IBD-related pathways by regulating critical gene expression

**Fig. 6:**
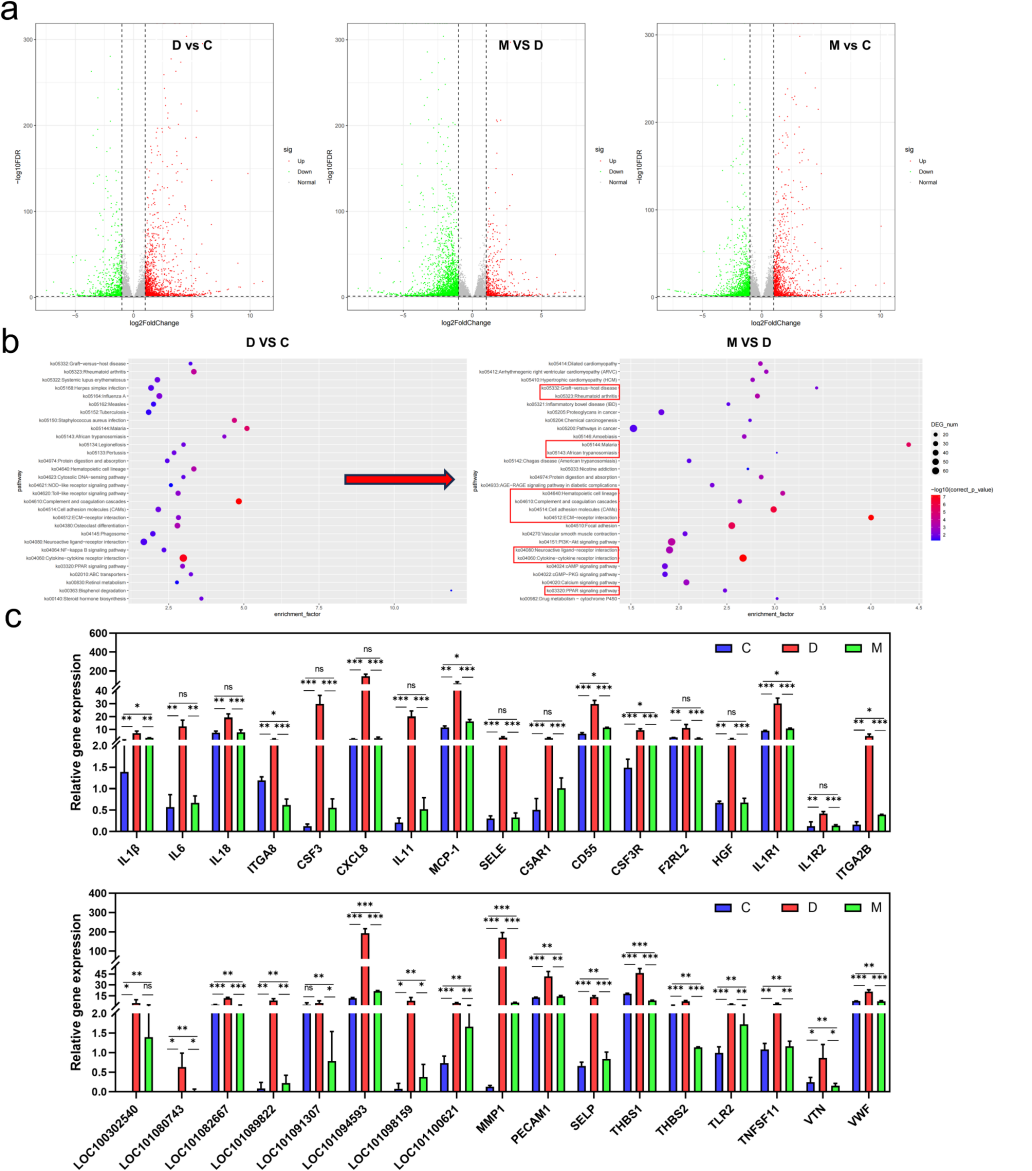
MSCs Modulate the Intestinal Transcriptome to Ameliorate Feline IBD-like Injury. (**a**) Volcano plot depicting differential gene expression; (**b**) Bubble chart illustrating KEGG enrichment analysis of differentially expressed genes; (**c**) Statistical analysis of differential gene expression. Significant variations had been denoted with * (*p* < 0.05), ** (*p* < 0.01), or *** (*p* < 0.001)

Differential gene expression analysis, facilitated by the R package DESeq2 and volcano plot visualization, identified significant up-regulation of 1,538 genes and down-regulation of 956 genes in response to DSS exposure compared to control conditions, with 639 genes down-regulated in MSC-treated samples (Fig. 6a). This comparative gene expression pattern indicates a convergence towards homeostasis in MSC-treated groups. KEGG pathway enrichment analysis of differentially expressed genes between DSS-exposed and control groups revealed enrichment of pathways associated with inflammation and immune function, such as cytokine-cytokine receptor interaction, Toll-like receptor signaling pathway (TLRs), and NF-kappa B signaling pathway (NF-κB), as well as affected protein digestion and absorption, potentially linking to DSS-induced intestinal damage (Fig. 6b). Intriguingly, 11 shared functional pathways were identified in the differential gene expression between MSC-treated and DSS-exposed groups, predominantly involved in the regulation of inflammation, immunity, and disease. Gene-pathway network mapping identified 131 genes co-regulated by DSS and MSC, with 36 critical genes implicated in multiple pathways subjected to statistical analysis (Fig. S4). These genes, encompassing cytokines, cell adhesion molecules, immunomodulatory factors, and those related to vascular function, cell signaling, proliferation, differentiation, tissue repair, and regeneration, were found to be significantly up-regulated after post-DSS exposure and subsequently suppressed by MSC treatment (Fig. 6c). This modulation of gene expression by MSCs presents a compelling rationale for their therapeutic potential in colonic injury induced by DSS.

### Correlation analysis

**Fig. 7:**
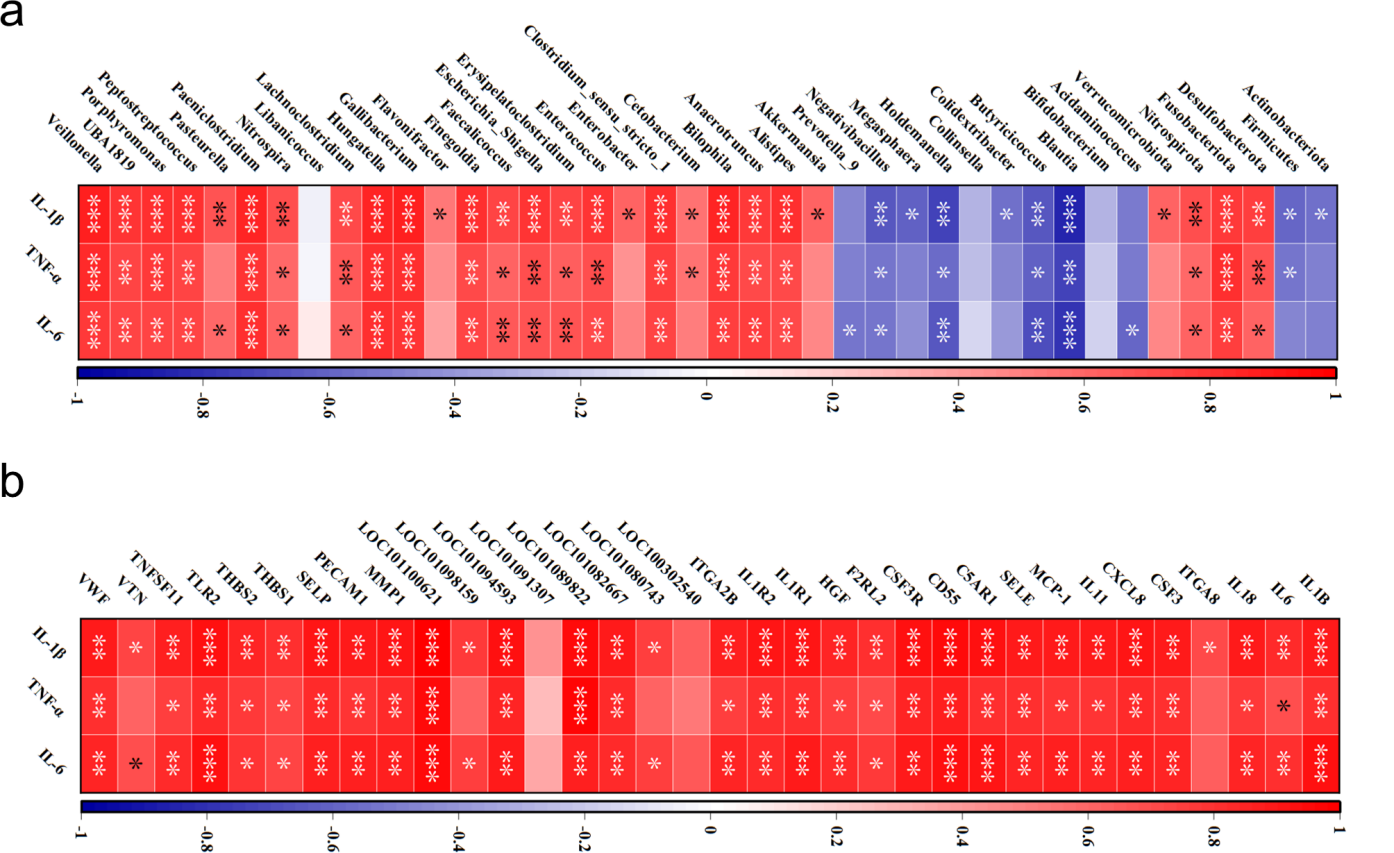
Correlation between Gut Microbiota, Intestinal Transcriptome, and IBD-like Injury. (**a**) Heatmap illustrating the correlation between inflammatory markers and gut bacterial taxa; (**b**) Heatmap depicting the correlation between inflammatory markers and differentially expressed intestinal genes. Significant variations had been denoted with the aid of * (*p* < 0.05), ** (*p* < 0.01), or *** (*p* < 0.001)

Previous findings suggest that the therapeutic efficacy of MSCs may be mediated through the correction of gut microbiota dysbiosis and altered gene expression profiles caused by DSS exposure. Pearson correlation coefficients were calculated, and correlation heat maps were graphically represented to examine the interplay between these key bacteria and regulatory genes in the context of DSS-induced injury and to clarify the underlying mechanisms of MSCs. The analysis revealed a significant correlation between bacteria with potential biological roles and the increased inflammatory responses induced by DSS (Fig. 7a). Notably, bacterial taxa that showed a positive correlation with host inflammatory status, such as Fusobacteriota, Desulfobacteriota, Peptostreptococcus, and Escherichia_Shigella, were found to be significantly upregulated following DSS exposure. Conversely, taxa that were inversely related to host inflammation, including Firmicutes, Actinobacteria, Bifidobacterium, and Blautia, were suppressed by DSS. These alterations were effectively counterbalanced by MSC intervention. Subsidiary data analysis indicated that genes presumed to have pivotal regulatory functions were positively correlated with the host’s inflammatory state (Fig. 7b). Prior data have established that DSS promotes the expression of these genes, while MSCs abrogate this response. Furthermore, robust correlations were identified between the bacteria of interest and the host genes (Fig. S5). These observations confirm that DSS exposure results in gut microbial imbalance and modifications in host gene expression, underscoring a significant interconnection between gut microbes and host genes, and highlighting the modulatory influence of MSCs on the therapeutic landscape.

## Discussion

IBD is an ensemble of idiopathic intestinal disorders, including CD and UC, that pose significant health and economic challenges globally, markedly diminishing the quality of life for affected individuals [15]. While IBD is prevalent among humans, it is also frequently observed in cats, a common domestic animal [16]. However, the majority of IBD research has been anthropocentric and murine-centric, resulting in a shortage of studies dedicated to feline IBD. The limited existing research has utilized DSS to replicate colonic injury in cats, similar to the induction of UC in mice; yet, these injuries have not undergone professional evaluation and have primarily served as a basis for comparing treated and untreated outcomes [17]. The current study builds upon these preliminary investigations by refining the DSS intervention protocol to ensure consistent induction of colonic injury. Concurrently, a professional pathologist authenticated the injury as characteristic of IBD without prior knowledge of the sample identity. Despite decades of recognition, IBD continues to present a formidable clinical challenge. Current clinical therapies aim to alleviate symptoms by reducing intestinal inflammation [18, 19]. Medications such as 5-aminosalicylic acid (5-ASA), dexamethasone (DEX), and methylprednisolone (MPS) are pivotal in IBD management, but their efficacy is not universal. Prolonged administration may also lead to severe adverse effects, including fatalities. It may increase the risk of infections and malignancies, potentially due to their irreversible depletion, rapid metabolism, and non-selective interactions [20, 21]. Emerging research suggests that MSCs, endowed with pluripotent differentiation potential and immune-modulatory properties, represent a promising therapeutic agent for treating IBD [22, 23]. A substantial body of research has demonstrated that MSCs can improve IBD-like lesions in mice, including the modulation of immune responses, the restoration of intestinal barrier function, and the enhancement of the gut microenvironment [13, 24, 25]. These studies provide a scientific rationale for the clinical application of MSCs. A comprehensive meta-analysis encompassing both animal and human studies has demonstrated that MSCs improve the disease status in UC mice and enhance the cure rate in clinical trials involving human subjects [26]. A study involving the intravenous administration of MSCs to seven IBD patients substantiated the safety and efficacy of this approach [27]. By innovatively modulating the expression of CX3C chemokine receptor 1 (CX3CR1) and IL-25, researchers have enhanced the immunosuppressive potential of MSCs for a more effective treatment of IBD [28]. Furthermore, a wealth of clinical and experimental data has informed a retrospective analysis corroborating the tissue reparative effects of MSCs in IBD and their safety in humans [29]. In concordance, the present study demonstrates the marked improvement of IBD-induced injury in cats through MSCs, evidenced by recovery of body weight, amelioration of fecal status, reduction in inflammatory markers, and restoration of intestinal barrier function, reinforcing the notion that MSCs offer a viable therapeutic strategy for IBD healing.

The interplay between the intestinal microbiota and IBD is characterized by complexity and intimacy [30]. A reduction in microbial diversity and alterations in community structure are explicitly associated with IBD pathology in affected individuals [31]. In this study, DSS significantly diminished the diversity of the intestinal microbiota and preferentially reduced the relative abundance of Firmicutes, a phylum known for its thick-walled characteristics. Concurrently, there was an increase in Ascomycetes, a shift indicative of dysbiotic microbial communities typical of IBD patients. Furthermore, the emergence of Fusobacteriota as a dominant flora post-DSS intervention suggests a profound alteration in community composition, further supporting the notion that DSS-induced injury shares similarities with IBD. The dysbiosis induced by DSS in this study was significantly alleviated following treatment with MSCs. Echoing these findings, a previous study has demonstrated that MSCs can restore the functionality of dysregulated microbiota, thereby treating Crohn’s disease in a murine model.

Additionally, it was observed that MSC treatment significantly elevated the Firmicutes-to-Bacteroidetes ratio (F/B). This metric correlates with systemic levels of short-chain fatty acids (SCFA) [32]. The beneficial role of SCFA in modulating intestinal inflammation is well-established, with recent research indicating that MSCs can enhance SCFA production derived from the gut microbiota, thereby recalibrating the intestinal mucosal Treg/Th2/Th17 balance and treating colitis [14]. Further in-depth analyses have revealed that DSS exposure leads to the overproliferation of certain pathogenic bacteria and a concomitant reduction in beneficial bacteria, potentially exacerbating intestinal inflammation and worsening IBD symptoms. For instance, DSS intervention was associated with a significant increase in Peptostreptococcus and Escherichia_Shigella and a marked decrease in Bifidobacterium, Megamonas, and Blautia. Peptostreptococcus, known for its selective enrichment in the fecal and mucosal microbiota of colorectal cancer patients, has been implicated in promoting colorectal cancer [33]. Escherichia_Shigella, a pathogen notorious for causing intestinal diseases, is prevalent in various intestinal disorders and is recognized as a significant contributor to colitis [34]. Bifidobacterium plays a pivotal role in maintaining intestinal microbiota balance by inhibiting harmful bacteria and fostering the growth of beneficial species [35].

Moreover, it enhances intestinal mucosal integrity and function, promotes mucosal repair and regeneration, and mitigates intestinal injury by inhibiting the TLR4/NF-κB and MAPK signaling pathways [36]. Megamonas, capable of fermenting a broad spectrum of carbohydrates, produce acetic acid, propionic acid, and lactic acid as end products [37]. Its relative abundance has been negatively correlated with the risk of colorectal polyps and is significantly diminished in the intestinal milieu of IBD patients, corroborating the findings of this study [38]. Blautia, known for its capacity to utilize hydrogen and carbon dioxide to produce acetate, supplies energy to intestinal epithelial cells, muscles, and brain tissue [39]. Furthermore, Blautia exhibits anti-inflammatory properties and prevents pathogen colonization through bacteriocin production [40]. It is significantly reduced in colorectal cancer patients, where it inhibits inflammation, promotes SCFA production, and maintains intestinal homeostasis [41, 42]. This highlights the therapeutic potential of MSCs in IBD, which may be mediated by promoting beneficial bacteria, inhibiting harmful bacterial growth, and enhancing SCFA concentrations in the gut.

Intestinal inflammation is characterized by complete interactions between the innate and adaptive immune systems, with cytokine dysregulation implicated in the progression and immunopathogenesis of IBD [43]. Damage to the intestinal epithelium allows inflammatory mediators to infiltrate the intestinal barrier, leading to tissue damage [44]. Our study observed significant alterations in the gene expression profiles of colonic tissues following DSS exposure. Subsequent KEGG functional enrichment analysis indicated a predominant impact on inflammation and immune function pathways. The cytokine-cytokine receptor interaction pathway is crucial in modulating the production and release of inflammatory mediators, influencing the migration and infiltration of inflammatory cells, and regulating the magnitude and duration of the inflammatory response [45]. Proinflammatory cytokines, including TNF-α, IL-1, and IL-6, activate downstream signaling pathways upon binding to their receptors, which enhances the inflammatory response [46]. DSS exposure also disrupts the ECM-receptor interaction pathway, essential for regulating the inflammatory response during extracellular matrix remodeling and cellular signaling, and impacts the adhesion, migration, and infiltration of inflammatory cells [47, 48]. The ECM-receptor interaction pathway has been shown to play a crucial role in recovery from enteritis in hybrid grouper, underscoring its importance in intestinal inflammation [49].

Additionally, alterations in ECM structure and function correlate with IBD. Furthermore, DSS exposure activates the TLR and NF-κB pathways, which are central to regulating the inflammatory response [50, 51]. TLRs, a class of pattern recognition receptors, initiate immune responses by identifying MAMPs and DAMPs, thereby triggering immune and inflammatory responses by activating signaling pathways [52]. The activation of TLRs can induce inflammatory responses by activating NF-κB, leading to the expression of genes associated with inflammation, such as IL-1β and TNF-α, as well as chemokines like CXCL8 [53]. Once activated, the NF-κB signaling pathway, can also upregulate TLR expression, creating a positive feedback mechanism that amplifies the inflammatory response [54]. Dysregulation of the TLR-NF-κB signaling pathway has been linked to various inflammation-related diseases, including rheumatoid arthritis, IBD, and systemic lupus erythematosus [55, 56]. Conversely, MSC treatment has been observed to modulate these key pathways, notably inhibiting the expression of inflammatory mediators and chemokines induced by DSS exposure. This aligns with previous findings indicating that MSCs suppress inflammation by enhancing extracellular matrix secretion and their capacity to scavenge pro-inflammatory factors [57]. MSCs are suggested to facilitate IBD primarily by enhancing local microcirculation, promoting tissue colonization and repair, modulating immune responses, and decreasing disease severity [58].

Furthermore, the therapeutic efficacy of MSCs derived from various sources for IBD is progressively being validated through scientific inquiry. Recent studies have demonstrated that MSCs harvested from human bone marrow exhibit notable neuroprotective and anti-inflammatory properties in a colitis model induced by 2,4,6-trinitrobenzenesulfonic acid (TNBS) [59]. Additionally, another study reports successful attenuation of inflammatory responses and a reduction in oxidative stress-induced tissue damage in experimental colitis through the localized injection of MSCs derived from human placenta [60]. Moreover, recent research has revealed that MSCs sourced from human umbilical cord have the potential to alleviate symptoms of experimental colitis by modulating the balance of the intestinal microbiota [24]. These investigations substantiate the emerging viability of MSC-based therapies for IBD. Notably, the study’s findings indicate that MSCs significantly reduce the CAM pathway, a pathway, which is heavily influenced by inflammatory signaling mechanisms. In states of inflammation, inflammatory mediators, such as cytokines and chemokines, can stimulate CAM expression, facilitating the adhesion and migration of leukocytes, thereby contributing to the accumulation of inflammatory cells and the propagation of the inflammatory response, exacerbating inflammation and tissue damage [61]. Evidence suggests that inhibiting CAM expression or function can effectively mitigate inflammatory responses, presenting a therapeutic opportunity for diseases with an inflammatory component [62]. Consequently, the pronounced suppression of CAMs by MSCs underscores their potential therapeutic impact in the context of IBD. The analyses presented herein illustrate that the deregulation of colonic inflammation after DSS exposure results in heightened expression of inflammation-associated genes, which can impair intestinal cell function, leading to intestinal barrier breakdown and a decline in nutrient absorption efficiency. Conversely, MSC treatment has been shown to inversely modulate DSS-activated inflammatory pathways, thereby inhibiting the expression of pro-inflammatory genes. Additionally, MSCs are posited to reduce the inflammatory response by enhancing the activation of anti-inflammatory pathways. This regulatory duality may constitute a pivotal mechanism underlying the therapeutic efficacy of MSCs in treating IBD.

## Conclusion

In the present study, we successfully induced intestinal injury and dysbiosis characteristics of feline IBD using the DSS model, thereby establishing a robust experimental framework for investigating the therapeutic efficacy of MSCs and delineating their underlying mechanisms of action. Our primary objective was to assess the potential therapeutic effects of MSCs in alleviating clinical symptoms of feline IBD and modulating the intestinal microbiota, as well as to explore the novelty of MSC-based approaches in IBD management. Comparative analysis between the DSS-induced IBD model and the MSC-treated cohort revealed that MSCs markedly alleviated DSS-induced intestinal inflammation, as evidenced by reduced inflammatory cell infiltration, diminished production of inflammatory mediators, and enhanced reparative processes of the intestinal mucosa. Moreover, MSCs exerted a significant modulatory influence over the intestinal microbiota, aiding in the reconstitution of the gut microbial balance disrupted by DSS, which is imperative for intestinal health and functionality. The findings of this study indicate that MSCs may exert their therapeutic effects through multiple mechanisms, including the inhibition of pro-inflammatory cytokine synthesis, facilitation of anti-inflammatory cytokine secretion, modulation of intestinal microbiota equilibrium, and reinforcement of intestinal barrier integrity. These insights unveil the therapeutic versatility of MSCs in IBD treatment and lay a scientific foundation for their clinical utilization. In conclusion, MSCs have demonstrated notable efficacy and safety profiles in treating feline IBD, suggesting their potential as an innovative therapeutic modality. Future work will focus on refining MSC treatment protocols and investigating their combinatorial effects with other therapeutic strategies, such as dietary interventions, antibiotic treatments, and immunosuppressive therapies, to create a more holistic and tailored treatment regimen for IBD sufferers.

## Electronic supplementary material

Below is the link to the electronic supplementary material.

Supplementary Material 1:Additional file 1 Fig.S1 Quality assessment of gutmicrobiota sequencing data. Fig.S2 Unweighted Pair-group Method with Arithmetic Mean (UPGMA) Analysis of Sample Phylogenetics. Fig.S3 Quality Assessment of Colonic Transcriptome Sequencing Data. Fig.S4 Gene-Functional Pathway Network Relationships. Fig.S5 Heat map of correlation between gutmicrobiota and hostintestinal gene expression profiles. Additional file 2. Professional pathology test reports. Additional file 3. Ethical approval of animal experiments. Additional file 4. Western blot results in a nun cropped view.

## Data Availability

The data that support the findings of this study are available from the corresponding author upon reasonable request, and the dataset presented in this study is available in the NCBI Sequence Read Archive (SRA) repository under accession number PRJNA1162671 (https://www.ncbi.nlm.nih.gov/bioproject/PRJNA1162671)
