# Supplementary material for: Mesenchymal stem cells: a novel therapeutic approach for feline inflammatory bowel disease
Source: Stem Cell Res Ther. 2024 Nov 9;15:409. doi: 10.1186/s13287-024-04038-y (PMC11550560; doi:10.1186/s13287-024-04038-y)
Supplement: Supplementary file 1 — Supplementary Material 1:Additional file 1 Fig.S1 Quality assessment of gutmicrobiota sequencing data. Fig.S2 Unweighted Pair-group Method with Arithmetic Mean (UPGMA) Analysis of Sample Phylogenetics. Fig.S3 Quality Assessment of Colonic Transcriptome Sequencing Data. Fig.S4 Gene-Functional Pathway Network Relationships. Fig.S5 Heat map of correlation between gutmicrobiota and hostintestinal gene expression profiles. Additional file 2. Professional pathology test reports. Additional file 3. Ethical approval of animal experiments. Additional file 4. Western blot results in a nun cropped view. [file 13287_2024_4038_MOESM1_ESM.pdf]

Supporting Information

Mesenchymal stem cells: a novel therapeutic approach for feline inflammatory bowel disease

Qiyun Xie[1†], Saisai Gong[1†], Jintao Cao[1] , Aoyun Li[3], Md. F. Kulyar[1], Bingyun Wang[2*]， Jiakui Li[1*]

[1]College of Veterinary Medicine, Huazhong Agricultural University, Wuhan, 430070, P.R. China

[2]School of Life Science and Engineering, Foshan University, Foshan, P.R. China

[3]College of Veterinary Medicine, Henan Agricultural University, Zhengzhou, P.R. China

*Correspondence

Bingyun Wang - School of Life Science and Engineering, Foshan University, Foshan, China; ORCID: 0000-0002-7472-1084; Email: bywang63@163.com

Jiakui Li - College of Veterinary Medicine, Huazhong Agricultural University, Wuhan, China; ORCID:0000-0002-6065-6648; Email: lijk210@mail.hzau.edu.cn

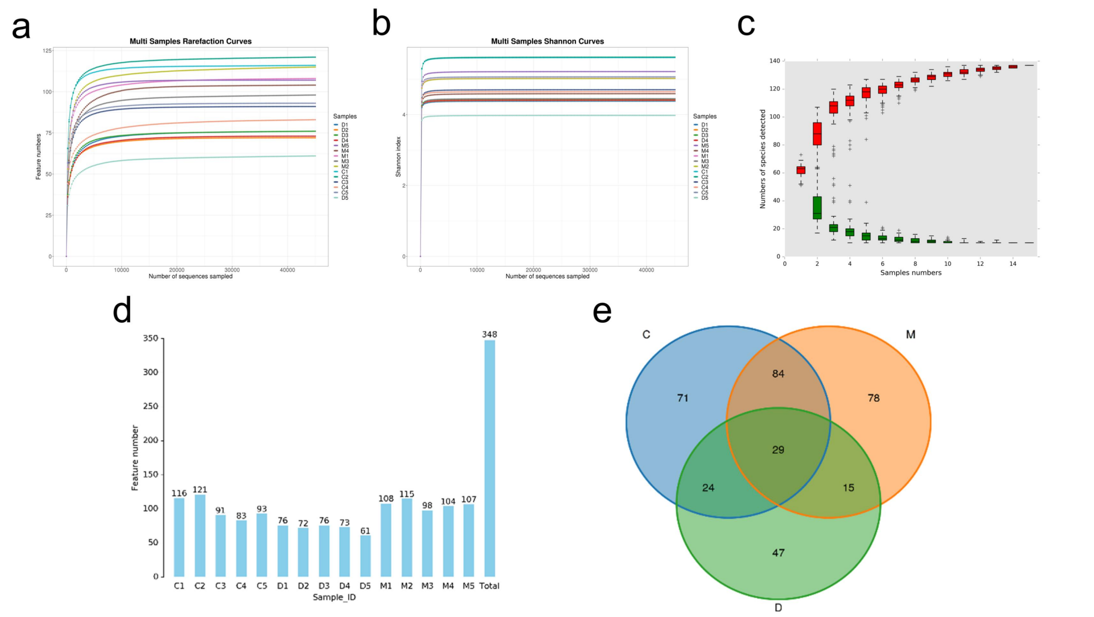

Figure S1. Quality assessment of gut microbiota sequencing data. (a) Rarefaction curves for estimating species richness; (b) Rank-abundance curves depicting species distribution; (c) Species accumulation curves for evaluating sampling adequacy; (d) Distribution of OTUs across samples; (e) Venn diagram illustrating the overlap of features among groups.

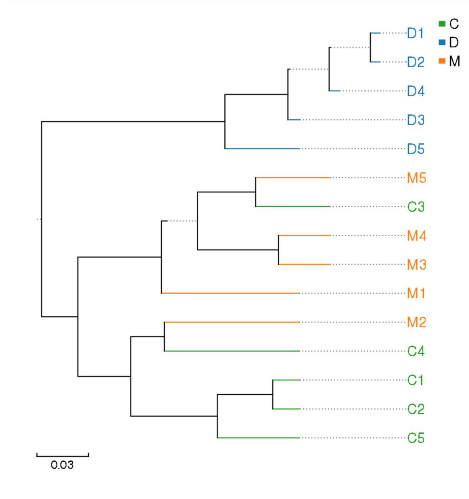

Figure S2. Unweighted Pair-group Method with Arithmetic Mean (UPGMA) Analysis of Sample Phylogenetics: Proximity of samples and brevity of branch lengths signify greater similarity in species composition。

a

sample_name	reads_number	reads_length	total_base	Q20_content(%)	Q30_content(%)	GC_content(%)
C1	43000256	150	6450038400	94	87	47
C2	37794674	150	5669201100	93	86	47
C3	39076430	150	5861464500	93	85	47
D1	48433202	150	7264980300	93	84	47
D2	52139200	150	7820880000	93	85	52
D3	46309566	150	6946434900	93	85	51
M1	40673326	150	6100998900	93	85	47
M2	59482248	150	8922337200	91	83	47
M3	36507238	150	5476085700	93	85	48

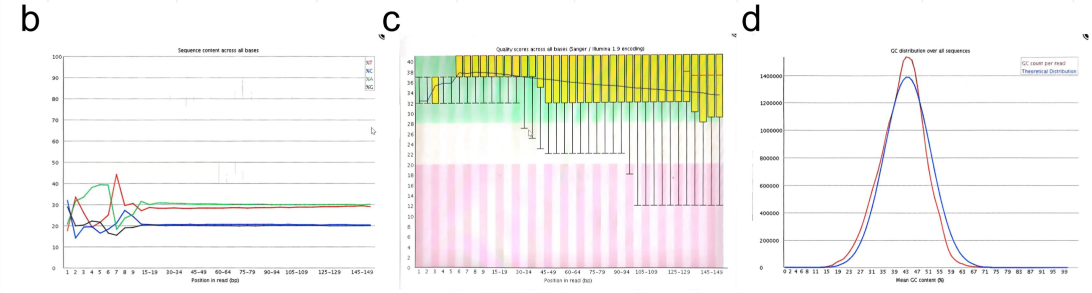

Figure S3. Quality Assessment of Colonic Transcriptome Sequencing Data. (a) Sequencing yield statistics; (b) Distribution of nucleotide composition in sequencing reads; (c) Representation of sequencing quality metrics; (d) Illustration of GC content distribution in sequencing data.

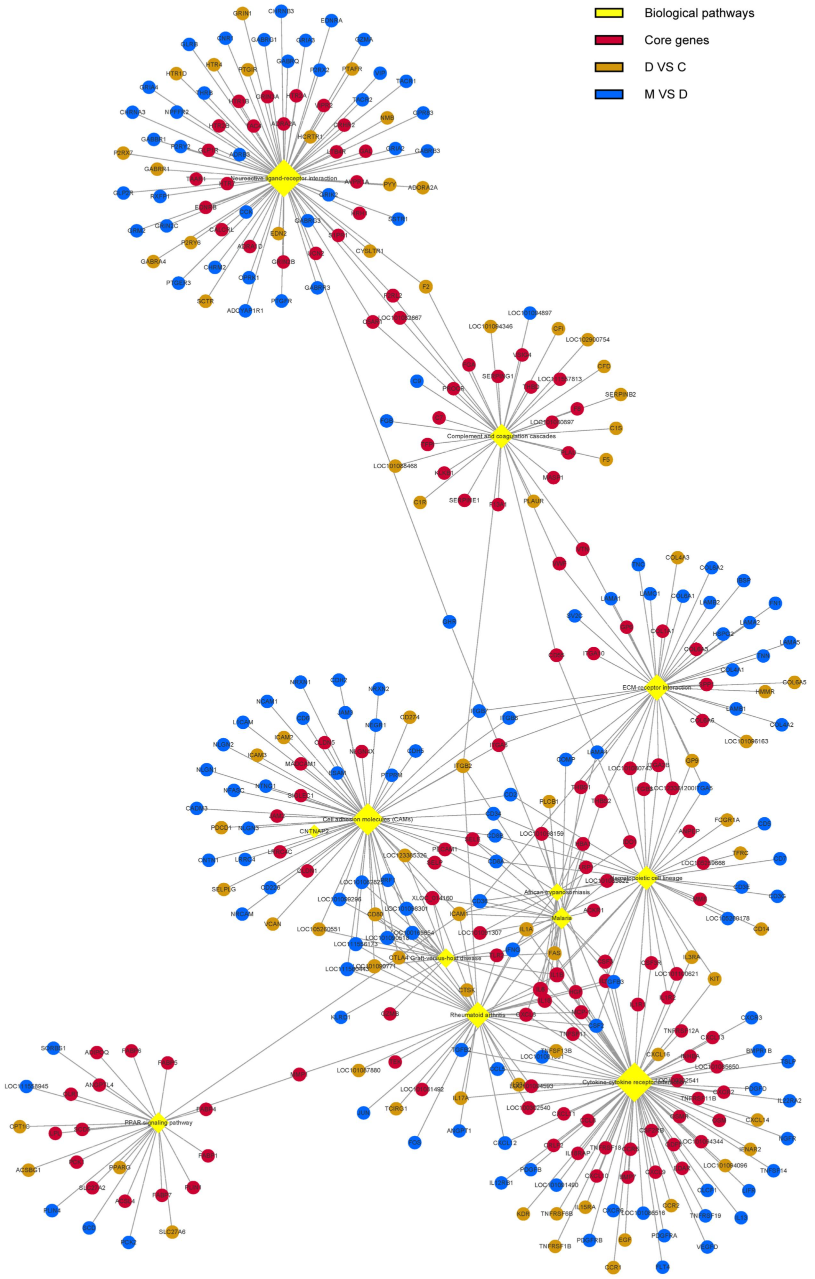

Figure S4. Gene-Functional Pathway Network Relationships.

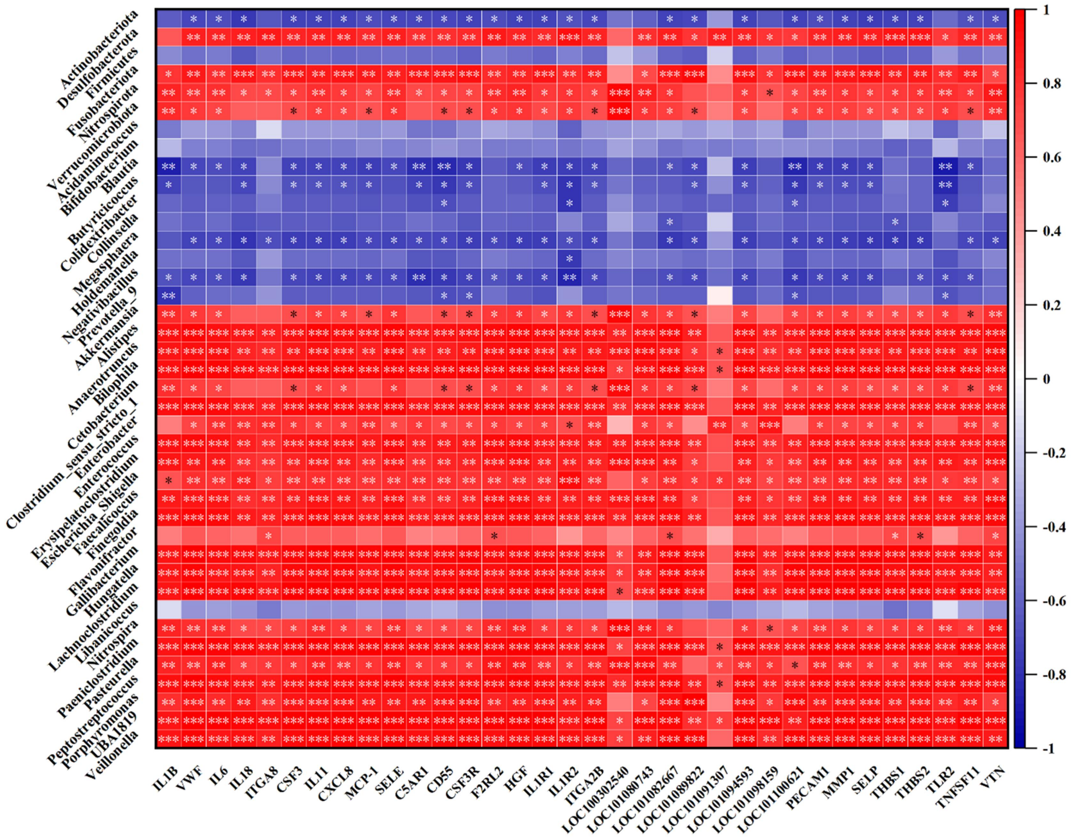

Figure S5. Heatmap of correlation between gut microbiota and host intestinal gene expression profiles.

病理报告书

SQ LAB 兽丘参考实验室 SQ Reference Lab

病例信息　　工单号：SH221110066

宠物名：	咪咪	病历编号：	NA（未提供）	性别：	母	是否绝育：	已绝育
物种：	猫	品种：	短毛	年龄：	1Y2M	宠主姓名：	NA（未提供）
采样日期：	2022.11.08	样本抵达日期：	2022.11.10	报告日期：	2022.11.15		
送检医院：	湖北-转诊中心（联合）-武汉-江汉			送检医师：	谢医师		
联系电话：	151****5635			电子邮箱：	X****@126.com		

样本：（数量、采样部位、形状、大小、颜色、质地）

结肠组织

过往病史：（临床症状、发病时长、发病部位）

NA（未提供）

组织病理学检查：

One section of colonic tissue reportedly biopsied from a spayed female cat are evaluated for microscopic pathology. The lamina propria of mucosa is variably expanded/infiltrated by lymphocytes, fewer plasma cells and rare neutrophils. Multifocally, thick mucoid substance overlies and fills the mucosal surface are crypts. The cell morphology of the epithelial cells of crypt and mucosal epithelium are unremarkable. The lymphatic vessels in the submucosa are variably dilated. No evidences of malignancy and infectious microorganisms are noted.

诊断：

1. **Colonic tissues (Figures 1-2):** Colitis, lymphoplasmacytic, chronic, diffuse, mild to moderate, with lymphangiectasia

   结肠组织：结肠炎、淋巴浆细胞性、慢性、弥漫性、轻微至中等、伴有淋巴管扩张

(本报告仅做病理诊断使用，不适用于法律诉讼相关使用)

总结:

The submitted colonic tissues exhibit lymphoplasmacytic colitis, which may raise the concern of inflammatory bowel disease (IBD) or other etiologies causing mucosal irritation. Differentiation between IBD and gastrointestinal lymphoma is a common diagnostic challenge that often requires more than histopathology. Lymphoma in the current case is less likely since the current lesions do not fit the criteria of lymphoma (cell morphology, low mitotic count, and no clusters of more than 5 intraepithelial lymphocytes or 5 adjacent epithelial cells overrun by lymphocytes). In addition to IBD, some cats with lymphoplasmacytic enteritis/gastritis are well responded to the treatment for dietary intolerance or hypersensitivity by a limited antigen diet. Therefore, controlled diet with prescription diet is recommended. Diet control for at least one month is necessary to rule-out the possibility of immune response to dietary antigens. Depending on the response, continue or change to another diet with a different protein source may be considered. Other etiologies leading to mucosal irritation include food allergy, intestinal dysbiosis, and parasites Therefore, fecal examination to rule-out parasitic infestation and diagnostic therapy by using probiotics to rule-out intestinal dysbiosis may be considered. In summary, food trial for dietary allergy/sensitivity and rule-out parasite/intestinal dysbiosis are recommended. If the condition is not improved, medical intervention for IBD and examinations for extragastrointestinal diseases are recommended.

*The cause of lymphangiectasia may be 1) lymphatic obstruction with increased lymphatic pressure and 2) increased vascular permeability associated with chronic enteritis/inflammatory bowel disease (IBD). Condition #2 is likely in this case.

1. 本病例结肠组织为淋巴浆细胞性炎症，需要考虑炎症性肠病（IBD）或其他造成黏膜刺激的病因。
2. 鉴别 IBD 和胃肠道淋巴瘤是相当大的挑战，有时会需要组织学之外的诊断工具辅助。本病例之病变不符合淋巴瘤的标准（细胞形态、低有丝分裂计数、没有超过 5 个上皮内淋巴细胞聚集或 5 个相邻上皮细胞都被淋巴细胞浸润），故须优先考虑 IBD。
3. 除了 IBD 外，部分患有淋巴浆细胞性胃炎/肠炎的猫只对低敏或无敏的饮食治疗反应良好；因此建议使用相关处方饲料进行饮食控制。控制的时间至少要一个月以上，这样才能排除对食物过敏的可能性。根据饮食控制期间的反应，可以考虑继续或转换成其他具有不同蛋白质来源的食物。
4. 其他造成黏膜慢性刺激的病因包含食物过敏、肠道菌丛失衡和寄生虫的刺激。可考虑进行粪检排除寄生虫，也可尝试给予消化道益生菌进行诊断性治疗。
5. 综合来说，建议针对饮食过敏/敏感进行低敏/无敏饮食，排除寄生虫、肠道菌丛失衡等病因、若无法改善则可开始针对 IBD 进行治疗/控制及须评估是否有其他消化道外的疾病存在。

*造成淋巴管扩张的常见的机制是：1）淋巴管阻塞，淋巴管压力上升；2)，慢性肠炎/炎症性肠病（IBD）导致的血管通透性上升。本病例可能与肠炎有关。

References

1. Gelberg, H. B. (2017). Alimentary System and the Peritoneum, Omentum, Mesentery, and Peritoneal Cavity. In: Pathologic Basis of Veterinary Disease (6th ed). J. F. Zachary (Ed). Mosby: 324-411.

Uzal, F. A., et al. (2016). Alimentary System. In: Jubb, Kennedy & Palmer's Pathology of Domestic Animals: Volume 2 (6th ed). M. G. Maxie (Ed). W.B. Saunders: 1-257.

实验室人员	病理兽医师
李鑫阳	台獸師字第6067號 病專獸字第0035號 李文達

对检测结果如有疑问，请于 5 个工作日内与本公司客服人员联系

审核：陳仕魁　　　　日期：2022.11.15

SQ REFERENCE LAB 报告章 Report Seal 兽丘参考实验室

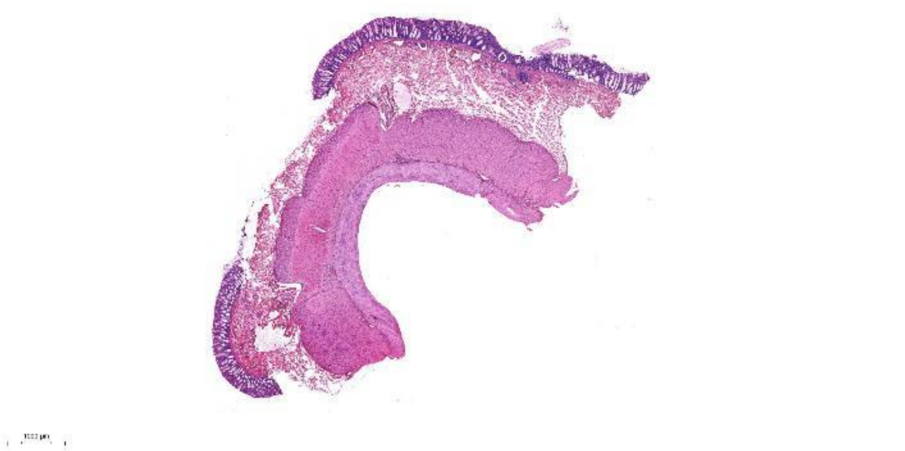	**Fig. 1** Photomicrography of submitted tissues with lymphangiectasia.
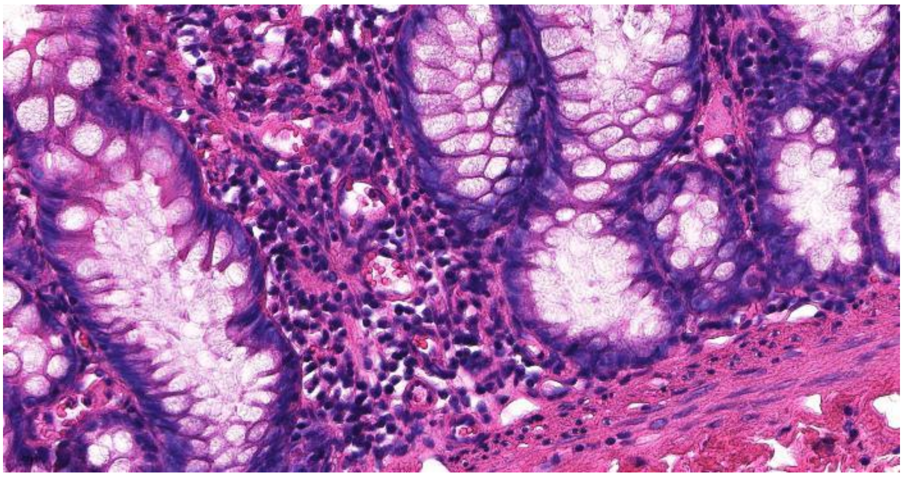	**Fig. 2** Photomicrography of lymphoplasmacytic infiltrations in the lamina propria.

(本报告仅做病理诊断使用，不适用于法律诉讼相关使用)

病理报告书

SQ LAB 兽丘参考实验室 SQ Reference Lab

病例信息

工单号：SH221110075

宠物名：	毛豆	病历编号：	NA（未提供）	性别：	母	是否绝育：	已绝育
物种：	猫	品种：	短毛猫	年龄：	1Y2M	宠主姓名：	NA（未提供）
采样日期：	2022.11.08	样本抵达日期：	2022.11.10	报告日期：	2022.11.16		
送检医院：	湖北-转诊中心（联合）-武汉-江汉			送检医师：	谢医师		
联系电话：	151****5635			电子邮箱：	xie****@126.com		

样本：（数量、采样部位、形状、大小、颜色、质地）

结肠组织

过往病史：（临床症状、发病时长、发病部位）

NA（未提供）

组织病理学检查：

Two sections of colonic tissue reportedly biopsied from a 1 year and 2 months old, spayed female cat are evaluated for microscopic pathology. The lamina propria of mucosa is variably expanded/infiltrated by lymphocytes, fewer plasma cells and rare neutrophils. Multifocally, thick mucoid substance overlies and fills the mucosal surface are crypts. The cell morphology of the epithelial cells of crypt and mucosal epithelium are unremarkable. The lymphatic vessels in the submucosa are variably dilated. No evidences of malignancy and infectious microorganisms are noted.

诊断：

1. **Colonic tissues (Figures 1-2):** Colitis, lymphoplasmacytic, chronic, diffuse, mild to moderate, with lymphangiectasia

   结肠组织：结肠炎、淋巴浆细胞性、慢性、弥漫性、轻微至中等、伴有淋巴管扩张

(本报告仅做病理诊断使用，不适用于法律诉讼相关使用)

总结:

The submitted colonic tissues exhibit lymphoplasmacytic colitis, which may raise the concern of inflammatory bowel disease (IBD) or other etiologies causing mucosal irritation. Differentiation between IBD and gastrointestinal lymphoma is a common diagnostic challenge that often requires more than histopathology. Lymphoma in the current case is less likely since the current lesions do not fit the criteria of lymphoma (cell morphology, low mitotic count, and no clusters of more than 5 intraepithelial lymphocytes or 5 adjacent epithelial cells overrun by lymphocytes). In addition to IBD, some cats with lymphoplasmacytic enteritis/gastritis are well responded to the treatment for dietary intolerance or hypersensitivity by a limited antigen diet. Therefore, controlled diet with prescription diet is recommended. Diet control for at least one month is necessary to rule-out the possibility of immune response to dietary antigens. Depending on the response, continue or change to another diet with a different protein source may be considered. Other etiologies leading to mucosal irritation include food allergy, intestinal dysbiosis, and parasites Therefore, fecal examination to rule-out parasitic infestation and diagnostic therapy by using probiotics to rule-out intestinal dysbiosis may be considered. In summary, food trial for dietary allergy/sensitivity and rule-out parasite/intestinal dysbiosis are recommended. If the condition is not improved, medical intervention for IBD and examinations for extragastrointestinal diseases are recommended.

*The cause of lymphangiectasia may be 1) lymphatic obstruction with increased lymphatic pressure and 2) increased vascular permeability associated with chronic enteritis/inflammatory bowel disease (IBD). Condition #2 is likely in this case.

1. 本病例结肠组织为淋巴浆细胞性炎症，需要考虑炎症性肠病（IBD）或其他造成黏膜刺激的病因。
2. 鉴别 IBD 和胃肠道淋巴瘤是相当大的挑战，有时会需要组织学之外的诊断工具辅助。本病例之病变不符合淋巴瘤的标准（细胞形态、低有丝分裂计数、没有超过 5 个上皮内淋巴细胞聚集或 5 个相邻上皮细胞都被淋巴细胞浸润），故须优先考虑 IBD。
3. 除了 IBD 外，部分患有淋巴浆细胞性胃炎/肠炎的猫只对低敏或无敏的饮食治疗反应良好；因此建议使用相关处方饲料进行饮食控制。控制的时间至少要一个月以上，这样才能排除对食物过敏的可能性。根据饮食控制期间的反应，可以考虑继续或转换成其他具有不同蛋白质来源的食物。
4. 其他造成黏膜慢性刺激的病因包含食物过敏、肠道菌丛失衡和寄生虫的刺激。可考虑进行粪检排除寄生虫，也可尝试给予消化道益生菌进行诊断性治疗。
5. 综合来说，建议针对饮食过敏/敏感进行低敏/无敏饮食，排除寄生虫、肠道菌丛失衡等病因、若无法改善则可开始针对 IBD 进行治疗/控制及须评估是否有其他消化道外的疾病存在。

*造成淋巴管扩张的常见的机制是：1）淋巴管阻塞，淋巴管压力上升；2)慢性肠炎/炎症性肠病（IBD）导致的血管通透性上升。本病例可能与肠炎有关。

References

1. Gelberg, H. B. (2017). Alimentary System and the Peritoneum, Omentum, Mesentery, and Peritoneal Cavity. In: Pathologic Basis of Veterinary Disease (6th ed). J. F. Zachary (Ed). Mosby: 324-411.
2. Uzal, F. A., et al. (2016). Alimentary System. In: Jubb, Kennedy & Palmer's Pathology of Domestic Animals: Volume 2 (6th ed). M. G. Maxie (Ed). W.B. Saunders: 1-257.

实验室人员	病理兽医师
李鑫阳	台獸師字第6067號 病專獸字第0035號 李文達

对检测结果如有疑问，请于 5 个工作日内与本公司客服人员联系

审核：陳仕魁　　　　日期：2022.11.16

SQ REFERENCE LAB 报告章 Report Seal 兽丘参考实验室

(本报告仅做病理诊断使用，不适用于法律诉讼相关使用)

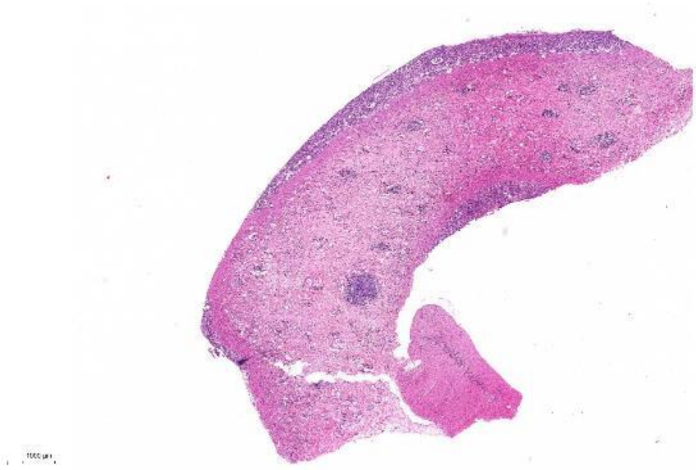	**Fig. 1** Photomicrography of submitted tissues with lymphangiectasia.
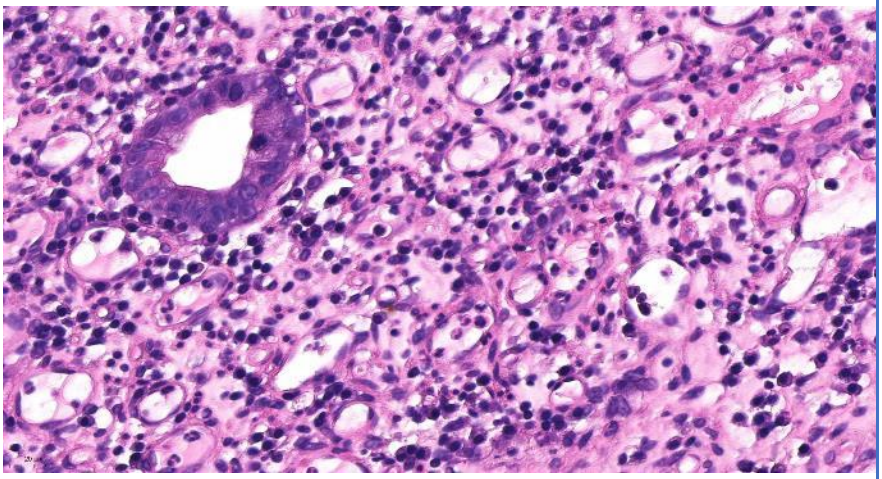	**Fig. 2** Photomicrography of lymphoplasmacytic infiltrations in the lamina propria.

Additional file 3

华中农业大学实验动物中心动物实验伦理审查表

The Tab of Animal Experimental Ethical Inspection of Laboratory Animal Centre, Huazhong Agriculture University

伦理编号(ID Number)：HZAUCA-2024-0026

<table>
<tr><td rowspan="12">申请人填写的相关信息(Related information filled by applicant)</td><td colspan="2">申请单位(Name of organization)</td><td colspan="5">华中农业大学动物医学院学院College of Veterinary Medicine, Huazhong Agriculture University</td></tr>
<tr><td colspan="2">项目负责人姓名(Name of project director)</td><td colspan="2">李家奎</td><td>项目负责人职称(Professional title)</td><td colspan="2">教授</td></tr>
<tr><td colspan="2">是否本单位做实验</td><td colspan="2">否</td><td>实验名称(Experiment title)</td><td colspan="2">间充质干细胞和肠菌移植治疗实验性猫炎症性肠病的探究Treatment of experimental feline inflammatory bowel disease with Mesenchymal stem cell and enterobacterial transplantation</td></tr>
<tr><td colspan="2">项目经费来源(Project sources)</td><td colspan="5">华中农业大学动物医院基金</td></tr>
<tr><td colspan="2">实验目的(Aim of experiment)</td><td colspan="5">研究间充质干细胞和肠菌移植对猫炎症性肠病的治疗效果To study the efficacy of Mesenchymal stem cell and Enterobacteriaceae transplants on cat inflammatory bowel disease</td></tr>
<tr><td colspan="2">实验动物使用许可证号(Number of the using of Laboratory Animal)</td><td colspan="5">SYXK（鄂）2020-0084</td></tr>
<tr><td rowspan="4">拟进动物情况</td><td>动物来源(Source of animal)</td><td colspan="5">华中农业大学实验动物中心 Laboratory Animal Centre, Huazhong Agriculture University</td></tr>
<tr><td>品种品系(Species or strain)</td><td>猫</td><td>等级(Grade)</td><td>无</td><td>规格(Specifications)</td><td>2-5公斤</td></tr>
<tr><td>数量(Number)</td><td colspan="2">42只 (♀21 只；♂21 只)</td><td>申请日期(Application date)</td><td colspan="2">2024-04-19</td></tr>
<tr><td>进驻日期(Entering date)</td><td colspan="2">2024-04-19</td><td>结束日期(Ending date)</td><td colspan="2">2024-10-19</td></tr>
<tr><td colspan="7">实验要点，包括实验方法、观测指标、实验结束后处死动物的方法等
(Outline of experiments, experimental methods, observational index, executing animal method, et. al)</td></tr>
<tr><td colspan="7"></td></tr>
</table>

	该研究本着实验动物福利和伦理的原则，本实验项目优化设计方案，严格计划动物需要数量，计划需要42只短毛猫，其中12只猫用于DSS诱导猫炎症性肠病的动物模型构建，15只猫用于间充质干细胞对实验性猫炎症性肠病的治疗效果评估，15只猫用于肠菌移植对实验性猫炎症性肠病的治疗效果评估。实验结束后，所有猫咪会进行绝育，找领养或放生。Based on the principles of animal welfare and ethics, the design of this experimental project was optimized. The number of animals needed was strictly planned, and the plan required 42 short-haired cats, twelve Cats were used to construct animal models of DSS-induced cat inflammatory bowel disease, and 15 cats were used to evaluate the therapeutic effects of Mesenchymal stem cell on experimental cat inflammatory bowel disease, 15 cats were used to evaluate the therapeutic effect of intestinal bacteria transplantation on experimental cat inflammatory bowel disease. At the end of the experiment, all cats will be sterilized, looking for adoption or release.			
	申请人签名 (Signature of applicant)		联系电话 (Telephone)	15102785635
	项目负责人签名 (Signature of Project director)		联系电话 (Telephone)	15102785635

声明(Statement)：

我将自觉遵守实验动物福利伦理原则，随时接受委员会的监督与检查，如违反规定，自愿接受处罚。

(I will conscientiously abide by the ethical principles of animal welfare, accept the supervision and inspection of the committee at any time, and voluntarily accept the punishment if any infringement.)

项目负责人签名(Signature of Project director)：

项目执行人签章(Signature of Project implementation)：

**审查依据** (Inspectioncontents)	1.该项目是否必须用实验动物进行实验，即能否用计算机模拟、细胞培养等非生命方法替代动物或用低等动物替代高等动物进行实验?<br>(Does laboratory animal must be used in the project? Could other methods such as computer simulation, cell cultivation or using the low-grade animal instead of the high-grade animal?)<br>2.表中所填申请人资格和所用动物的品种品系、质量等级、规格是否合适，能否通过改良设计方案或用高质量的动物来减少所用动物的数量?<br>(Are the qualification of applicant，species or strain, grade and specifications of animals suitable? Could the quantity of animals be reduced by improving the study design or using high quality animals?)<br>3.能否通过改进实验方法、调整实验观测指标、改良处死动物的方法，来优化实验方案、善待动物?<br>(Could the study design and animal treatment be refined by ameliorating experimental method, adjusting observational index, executing animal method?)

**专家审查** (Professor inspection)	专家意见 (Suggestion of Professor)	专家签名 (Signature of Professor)	日期 (Date)
	同意		2024年 05 月 07日
	同意		2024年 05 月 07日

<table>
<tr><td colspan="2">科学伦理委员会负责人签名(Signature of Ethics Committee Director)：
赵书红
2024年 05 月 08日</td></tr>
<tr><td>审查结果

(是否同意申请人的实验方案)
(Results of inspection)</td><td>伦理委员会意见

(Attitude of the Ethics Committee)：
同意

2024年 05 月 08日</td></tr>
</table>

Additional file 4

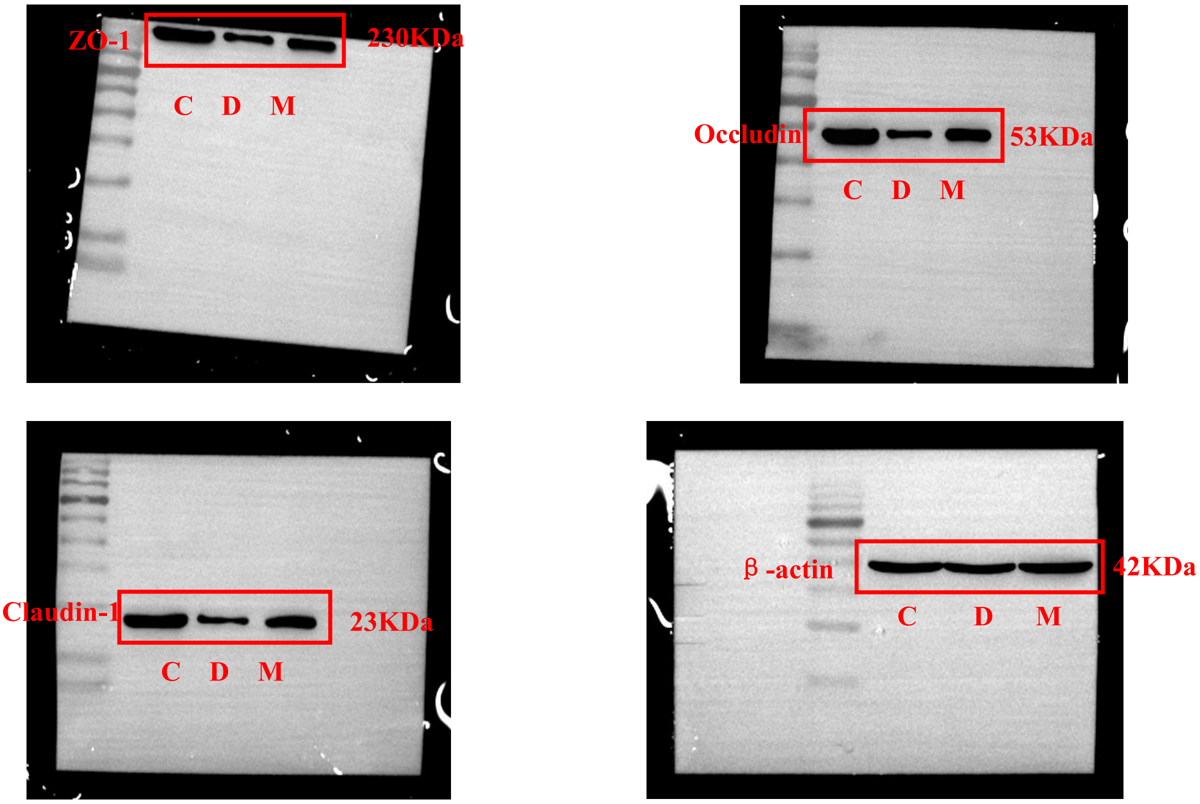

Additionalfile 4. Western blot results in an uncropped view.
